# The dynamics of brain T cell populations during the course of rasmussen encephalitis: from expansion to exhaustion

**DOI:** 10.1186/s12974-025-03477-5

**Published:** 2025-06-12

**Authors:** Katharina M. Mair, Victoria Guggenberger, Laia Verdú de Juan, Ulrike Köck, Hans Lassmann, Roland S. Liblau, Christian G. Bien, Jan Bauer

**Affiliations:** 1 Department of Neuroimmunology, Centre for Brain Research, Medical University of Vienna, Vienna, A-1090 Austria; 2 Institute for Inflammatory and Infectious Diseases, INSERM UMR1291 - CNRS UMR505, Toulouse, France; 3 Department of Epileptology, Krankenhaus Mara, Bethel Epilepsy Center, Medical School OWL, Bielefeld University, Bielefeld, Germany

**Keywords:** Rasmussen encephalitis (RE), Neuroinflammation, Neurodegeneration, Tissue resident memory T cells (T_RM_), γδ T cells, Immune checkpoint molecules

## Abstract

Rasmussen Encephalitis (RE) is a chronic, unilateral epileptic disorder mostly found in children. Neuropathologically, it is characterized by T lymphocyte infiltration targeting neurons and leading to microglia activation, astrogliosis, and cortical degeneration. Within a patient’s brain, distinct pathological stages are found that offer a unique opportunity to study T cell dynamics in situ. Using quantitative multiplex fluorescence imaging, we analyzed CD103^+^ and CD69^+^ Tissue-resident memory T cells (T_RM_) across different disease stages. This analysis revealed that T_RM_ were more abundant in the parenchyma than in the perivascular space, suggesting that their differentiation occurs locally after antigen encounter. Further, part of the T_RM_ expressed Granzyme-B (GrB) and frequently were attached to neurons, suggesting that they are actively involved in neuronal destruction. While T_RM_ showed a stage-dependent increase in older lesions, the proportions of these cells did not correlate with disease duration, indicating that their accumulation may be more dependent on the local environment in the lesion than on the length of the disease. In addition, we found that T cells using the γδ T cell receptor comprised up to 66%. Like CD8^+^ T cells, the γδ T cells could develop a T_RM_ phenotype and, while expressing GrB^+^ granules, they were seen attached to neurons, suggesting that they are involved in neuronal destruction. Finally, analysis of exhaustion- and T_RM_-associated immune checkpoint control markers PD-1 and LAG-3 revealed a significant stage-dependent increase in PD-1 expression in the oldest lesions. In contrast, LAG-3 expression did not show any stage-specific pattern, pointing towards a distinct regulatory mechanism. The study demonstrates a dynamic and one-way T cell response throughout the course of RE at a given spot in the CNS: from the establishment of T cell residence after entry into the CNS, the killing of neurons, and eventually T cell exhaustion. It further suggests an important role of γδ T-cells in the propagation of disease and lesions.

**Supplementary Information:**

The online version contains supplementary material available at 10.1186/s12974-025-03477-5.

## Introduction

Rasmussen encephalitis (RE) is a rare but severe neurological disease, that predominantly affects young children [1–3]. This puzzling condition, which is characterized by chronic inflammation and neuronal loss resulting in neurologic deficits, including epilepsy, is typically limited to only one brain hemisphere [4–6]. The cause of RE is still unclear. Neuropathologically, there is a great diversity of lesions within the brains of RE patients. Based on T cell infiltration, microglia activation, neuronal loss, and astrocyte gliosis, Pardo et al. defined five stages, noting that all stages can be present within one hemisphere in one single patient [4]. Stage 0 is considered the normal-appearing cortex. Stage 1 is characterized by discrete foci of lymphocytes, with no or minimal evidence of neuronal injury. Our own histopathological studies revealed that within otherwise normal appearing cortical areas, small microglial nodules (described as primary microglial nodules) provide an environment for T cells as an initiating step for the inflammatory response. This inflammatory environment is further characterized by increased levels of interferons and other pro-inflammatory cytokines and chemokines [7]. As the disease progresses, T-cell infiltration increases, marking the transition to stage 2. During this stage, neuronal injury and degeneration become apparent. Stage 3 is defined by increased neuronal loss to a point where the brain shows cortical atrophy. In stage 4, neuronal loss culminates and results in extensive destruction of the cerebral cortex.

At present, cytotoxic T cells are considered the primary cause of neuronal destruction. A resulting imbalance of inhibitory and excitatory neurons most likely contributes to the generation of epileptic seizures. The majority of the infiltrating T cells are CD8^+^ cytotoxic T cells that contain Granzyme-B (GrB). Those T cells can be observed in close apposition to neurons, exhibiting directed release of their cytolytic granules towards them. Collectively, these results suggest neuronal death in a GrB-dependent manner [8–10]. Further studies performed in mouse models have provided an understanding of the role of T cells in RE. Immune-deficient NSG mice that received peripheral blood mononuclear cells from patients diagnosed with RE developed severe seizures, astrogliosis, and accumulation of human T cells in the brain [10]. Moreover, spectra-typing of T cells from human RE brain lesions indicated that T cells expand from antigenic epitope-responding precursor T cells, suggesting an antigen-driven immune response [9, 11]. However, no target antigen in RE has been identified yet. Besides the conventional αβ^+^ T cells, Owens et al. and Al Nimer et al. described the presence of γδ^+^ T cells in RE. These cells are known for their ability to respond to a wide range of antigens and contribute to tissue inflammation [12, 13]. In contrast to αβ^+^ T cells, the γδ^+^ T cells recognize antigens in an MHC independent manner [14], with the ability to detect a broad range of molecules, including non-peptidergic antigens [15]. However, their exact role in RE, whether detrimental or protective, their prevalence during disease progression, and further phenotypical characteristics remain unclear. Although cytotoxic T cells appear as key drivers in neuronal loss, the phenotype and the dynamics of these cells throughout the disease progression are not well understood.

Recently, the scientific community has shown a significant increase in interest in tissue-resident memory T cells (T_RM_) due to their central role in various immune responses. Recent studies suggest that T_RM_ are a driving force in disease progression by contributing to the maintenance of inflammation and neurodegeneration [16]. In the central nervous system, most T cells, present in the normal brain and in neurodegenerative conditions, are T_RM_ [17] and are abundant in conditions of chronic inflammation [18]. Due to their compartmentalized nature, in the brain parenchyma behind the blood-brain barrier (BBB), T_RM_ might evade the effects of drugs targeting circulating T cells. This is supported by the finding that T_RM_ can sustain brain damage independent of T cells in the circulation [19]. In parallel, T-cell exhaustion due to persistent antigenic stimulation is a key feature of chronic viral infections and cancer [20]. Typically, these exhausted cells start to express checkpoint inhibitor molecules, such as PD-1, CTLA4, and LAG-3 [21, 22]. These molecules are also highly expressed by T_RM_ isolated from multiple solid tumor types [23]. Checkpoint inhibitor therapy is an exciting new way to battle cancer. However, it has also led to an increase in paraneoplastic encephalitis with antibodies directed against a large number of antigens [24–29]. Like RE, these paraneoplastic diseases are mediated by cytotoxic T cells [30–32]. It becomes apparent that the immune dynamics in cytotoxic T-cell-mediated encephalitis require further exploration to shed light on their complexity. The broad spectrum of lesions, along with the massive infiltration of T cells as the disease progresses, allows to study T cell dynamics in RE. A deeper understanding of the presence of T_RM_, the induction of exhaustion of these T cells, and the role of γδ^+^ T cells in RE provides insight into their contribution to tissue damage for patients diagnosed with RE and possibly also for other CD8^+^ T cell-mediated brain diseases.

## Patients and methods

### Patients and sample selection

This study was performed on formaldehyde-fixed and paraffin-embedded (FFPE) surgical specimens from the Bethel Epilepsy Center, Bielefeld, Germany, between 1991 and 2015. The diagnosis of encephalitis compatible with Rasmussen Encephalitis (RE) was made by board-certified neuropathologists; the diagnosis finally rested upon the European consensus criteria [33]. In total 414 blocks from 42 RE patients were analyzed. Staging on these blocks was performed according to the criteria from Pardo et al. based on Iba1, CD3, HLA-DR, GFAP, and NeuN staining [4]. We aimed to include a broad range of samples, with as many patients presenting all 4 pathological stages. In Pardo´s classification, the lesions are divided into 4 stages, although in reality the lesion progression is fluent. We selected cortical samples that best fit Pardo´s criteria of the various stages. However, since stage 2 in Pardo´s semi-quantitative classification is very heterogeneous in terms of both inflammation and neuronal loss, we, for this stage 2, selected samples that were homogenous with respect to degree of inflammation and neuronal loss and noticeably were in between stage 1 (only local inflammation and no apparent neuronal loss) and stage 3 (clear presence of spongiosis). This means that blocks with criteria closer to stage 1 (mild inflammation and little neuronal loss) or stage 3 (those with strong inflammation and severe neuronal loss but without spongiosis) were excluded. Omitted were also blocks with non-cortical (hippocampal) specimens or blocks with artefactual changes such as resection-induced bleedings. Since stages 3 and 4 were rather rare in our sample of surgical resections, we combined these two stages into a single group (stage 3–4). Due to these criteria, the final sample size was reduced to 39 blocks from 16 patients. For one patient (RE13, Table 1), we used blocks from the first surgery as well as from the resection 3 years later. Furthermore, eight samples from deceased patients without any neurological conditions were included as controls. (Table 1).

**Table 1:** Patient demographic data, clinical stages, and neuropathological analysis

**ID**	Sex	Disease Duration*	**Stage available**	Age at Surgery/ Death (yrs)	Hemisphere/	Multiplex protocols used per block/stage		
		(yrs)			**Location**	Stage 1	Stage 2	Stage 3–4
**RE1**	F	**0.9**	**1, 2**	7.9	l	**γδ T**_RM_ **T**_EX_	**# γδ T**_RM_ **T**_EX_ **GrB**	(n. a)
**RE2**	M	**0.9**	**3**	6.4	r	(n. a)	(n. a)	**γδ** **T**_EX_
**RE3**	M	**1**	**1, 2, 3**	4.8	r	γδ **T**_EX_	**#** γδ **T**_RM_**T**_EX_	**γδ T**_RM_ **T**_EX_
**RE4**	F	**1.2**	**1, 2**	5.2	r	**γδ T**_RM_ **T**_EX_	**γδ T**_RM_ **T**_EX_ **GrB**	(n. a)
**RE5**	F	**1.5**	**1, 2**	13.5	l	**γδ** **T**_EX_	**γδ T**_RM_ **T**_EX_ **GrB**	(n. a)
**RE6**	F	**1.7**	**3**	8.3	l	(n. a)	(n. a)	**γδ** **T**_RM_ **T**_EX_ **GrB**
**RE7**	F	**2.2**	**1**	7.2	l	**γδ** **T**_EX_	(n. a)	(n. a)
**RE8**	F	**2.5**	**1, 2, 3**	12	r	**γδ T**_RM_ **T**_EX_	**γδ T**_RM_ **T**_EX_ **GrB**	**# γδ T**_RM_ **T**_EX_
**RE9**	M	**3.5**	**1, 2**	4.5	r	**γδ** **T**_EX_	**γδ** **T**_EX_	(n. a)
**RE10**	M	**3.7**	**1, 2,3**	34.2	r	**γδ** **T**_EX_	**T**_EX_	**γδ T**_RM_ **T**_EX_
**RE11**	M	**5.5**	**1, 2, 3**	33.5	l	**γδ** **T**_EX_	**γδ T**_RM_ **T**_EX_	**γδ T**_RM_ **T**_EX_
**RE12**	M	**5.7**	**3**	11.7	r	(n. a)	(n. a)	**γδ** **T**_EX_
**RE13**	M	**5.7**	**1, 2, 3**	18.1	r	**# γδ T**_EX_	**γδ** **T**_EX_	**# γδ** **T**_EX_
Resection:		**9.7**		21.3				
**RE14**	M	**10.9**	**3**	10.9	l	(n. a)	(n. a)	**# γδ**
**RE15**	F	**17.3**	**1, 2, 3**	29.3	l	**γδ** **T**_EX_	**γδ** **T**_EX_	**γδ** **T**_EX_
**RE16**	M	**18.3**	**1, 2, 3**	29.3	r	**γδ** **T**_RM_ **T**_EX_	**γδ** **T**_RM_ **T**_EX_ **GrB**	**γδ** **T**_RM_ **T**_EX_
**CO1**	F	-	**-**	65.5	-	**CD3**		
**CO2**	F	-	**-**	n.d	-	**CD3**		
**CO3**	F	-	**-**	67.6	-	**CD3**		
**CO4**	M	-	**-**	63.8	-	**CD3**		
**CO5**	M	-	**-**	72	-	**CD3**		
**CO6**	F	-	**-**	48.7	-	**CD3**		
**CO7**	M	-	**-**	63.8	-	**CD3**		
**CO8**	M	-	**-**	45.4	-	**CD3**		

Overview of Rasmussen Encephalitis (RE) patients and Controls (CO). This table provides a breakdown of the histopathological stages available for each patient alongside disease duration and the age of surgery for RE patients or death for Controls. The multiplex protocols used for quantification are outlined on the left: multiplex protocols for γδ T cells, T cells with resident memory markers (T_RM_), T cells with exhaustion markers (T_EX_) and Granzyme B expressing T cells (GrB) were applied (for further details see Suppl. Table 2). Abbreviations: CD3 = single staining for CD3 was performed; RE = Rasmussen Encephalitis F = female; M = male; n.a. = not available; l= left; r = right; *Disease duration equals the start of Epilepsy; #two blocks were available.

## Immune histopathological evaluation

RE samples were analyzed by (double) labeling with Iba1 and CD3, Iba1 and HLA-DR as well as NeuN and GFAP. These stainings were performed according to the previously described protocol [34]. In summary, sections were dewaxed and steamed in a conventional household food steamer to achieve antigen retrieval. The primary antibodies (Supplementary Table 1) were applied overnight at 4℃. Then, incubation with the secondary biotinylated antibody (targeting CD3, HLA-DR, and NeuN, respectively) as well as the secondary peroxidase-conjugated antibody (targeting Iba1 and GFAP, respectively) was applied and followed by incubation with peroxidase-conjugated streptavidin for 1 h at room temperature (RT). To increase sensitivity, anti-CD3 and anti-NeuN were performed with tyramide enhancement [34]. CD3, HLA-DR, and NeuN staining were developed with Fast Red, and Iba1 and GFAP were developed with Fast Blue. As Iba-1 and CD3 both originate from rabbit and NeuN and GFAP derive from mouse, an additional steaming heat-induced epitope retrieval (HIER) was performed after Catalyzed Signal Amplification (CSA) enhancement, before the application of HLA-DR and GFAP to prevent cross-recognition by the antibodies from the same species. The sections were scanned with a slide scanner (NanoZoomer Digital Pathology, Hamamatsu Photonics) at x200 magnification and analyzed with the Hamamatsu NDPI viewer (https://www.hamamatsu.com/eu/en/product/life-science-and-medical-systems/digital-slide-scanner/U12388-01.html).

## Multiplex immunofluorescence labelling

A detailed list of the analyzed combinations of antibodies is presented in Supplementary Table 2. The following protocol was used throughout all stainings; labeling for markers of interest was performed using an Akoya Fluorescent Multiplex kit according to the manufacturer’s protocol. In brief, sections were steamed in antigen retrieval buffer pH 9.0 (AR9) or citrate for 60 min in a household food steamer (Braun), followed by a 10-minute blocking step with Opal Antibody Diluent/Block solution (Akoya Biosciences, Marlborough, USA). Thereafter, the primary antibodies were incubated for 2 h at RT or overnight at 4 °C. Following a washing step with Tris-buffered saline with Tween20, the secondary antibody (HRP conjugated) was introduced for 30 min at RT. Subsequently, the fluorophores (Opal 480, Opal 520, Opal 570, Opal 620, Opal 690, or Opal 780) were implemented. Before applying the next primary antibody, the sections were fixed with 4% paraformaldehyde for 10 min at RT, followed by another round of antigen retrieval step using AR6 for 30 min. Ultimately, the nuclei were stained with 4′,6-diamidino-2-phenylindole (DAPI).

## Cell quantification

To quantify multi-labeled cells, fluorescent stainings were scanned with the Vectra Polaris Automated Quantitative Pathology Imaging System from Perkin Elmer and quantified semi-automatically with QuPath software according to the online manual [35]. This software offers a tool for machine learning-based cell quantification. To this end, for every slide, the image was split into the respective channels. Next, cell detection was performed based on DAPI staining, and the object classifier was trained and validated manually. Eventually, an area of interest (0.09–68.31 mm²) was selected, and the respective classifiers were applied.

### Statistical analysis

For comparisons involving more than two groups, we employed the Kruskal-Wallis test followed by Dunn’s multiple comparison. For two-group comparisons, the Wilcoxon test was employed. Correlations were assessed with Spearman’s rank correlation coefficient. p-values below 0.05 were considered statistically significant. All statistical analyses were performed using GraphPad Prism 6.

## Results

### Different T cell subsets are found throughout all pathological stages

**Fig. 1:**
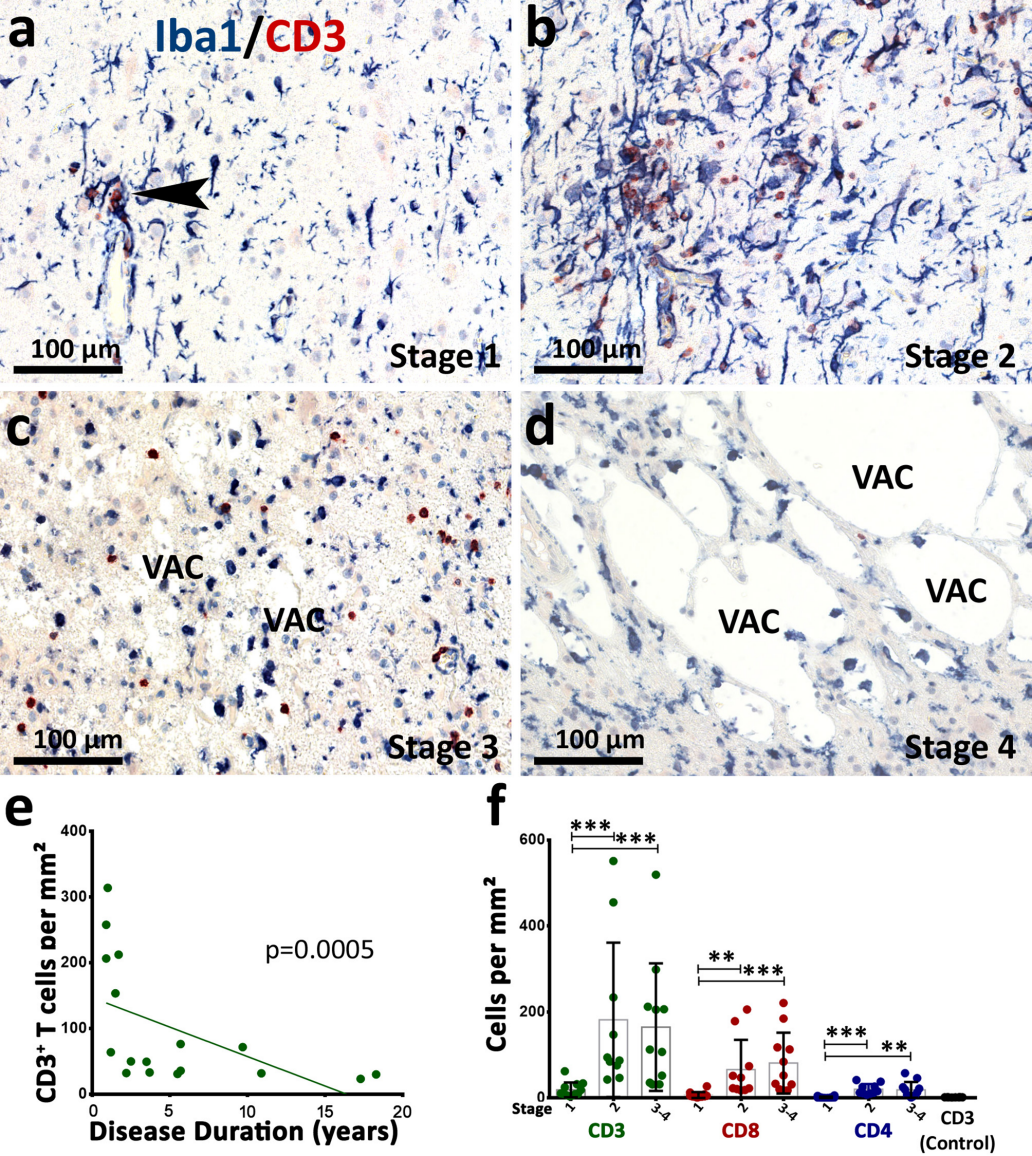
Different stages within Rasmussen encephalitis brains. (**a-d**) IHC double-labeling was performed for the characterization of CD3^+^ T cell infiltration and Iba1^+^ microglia across different RE stages: (**a**) In stage 1, discrete local CD3^+^ T cell infiltration (arrowhead) is observed. (**b**) In stage 2, an increase of CD3^+^ T cell infiltration accompanied by more pronounced microglial activation can be seen. T cells are now present all over the cortical grey matter. (**c**) In stage 3, neuronal loss becomes visible by the presence of small vacuoles (VAC). (**d**) Neuronal loss and spongiosis are even more pronounced in stage 4. (**e**) Stage-independent quantification of CD3^+^ T cells in RE patients. The analysis shows that the number of CD3^+^ T cells declines with longer disease duration (Spearman correlation test: *p* = 0.0005, *r*= -0.7669). (**f**) Shows T cell numbers of different (CD3, CD8, and CD4) T cell subsets across the stages of RE, with CD3^+^ T cell infiltration in control samples for reference. A strong increase of all T cell subtypes is seen between stage 1 and stage 2 and 3–4. Data are presented as median with interquartile range; (**e**): Spearman rank correlation was performed; (**f**): Kruskal-Wallis with post hoc Dunn’s multiple comparison test was performed, asterisks represent the result of post hoc multiple comparison: ***p ≤ 0.001, **p ≤ 0.01

As described in the materials and methods, we selected 39 blocks with different stages from 16 patients (Table 1; Fig. 1a-d). The selected blocks showed significant variability in the numbers of infiltrating CD3^+^ T cells, ranging from 4.1 to 551.3 cells/mm² (median 52.0 cells/mm²), whereas controls without neurological disease show a median number of 1.6 CD3^+^ T cells/mm². We found that there is a significant decrease in CD3^+^ T cell density with ongoing disease duration (Fig. 1e). Furthermore, stage-specific analysis showed a significant increase of CD3^+^ T cells in stage 2 and stage 3–4 compared to stage 1 (Fig. 1f). No significant difference was observed between stages 2 and 3–4, which contradicts the findings of Pardo et al. [4]. CD8^+^CD3^+^ cytotoxic T cells comprise a median number of 47.46% of all CD3^+^ T cells in the parenchyma. Similar to the CD3^+^ T cell numbers, the numbers of infiltrating CD3^+^CD8^+^ cytotoxic T cells increased significantly from stage 1 to stage 2 and to stage 3–4 (Fig. 1f). The number of infiltrating CD4^+^CD3^+^ T cells ranged from 0 to 57.7 cells/mm² (median 7.4 cells/mm²) and comprised a median number of 11.87% of all CD3^+^ T cells. In general, CD4^+^CD3^+^ T cells were less infiltrating the brain parenchyma in comparison to CD8^+^CD3^+^ T cells and were mostly confined to the perivascular space (not shown). Like CD3^+^ and CD8^+^ T cells, CD4^+^ T cells increased in numbers in stage 2 and stage 3–4 as compared to stage 1 (Fig. 1f). There was no change in the proportion of CD8^+^ and CD4^+^ T cells of all CD3^+^ T cells with ongoing disease duration (Supplementary Fig. 1a).

## Stage-specific analysis: Tissue-resident memory T cells are more abundant in older lesions

### T_RM_ increase with ongoing lesion progression but not disease duration

**Fig. 2:**
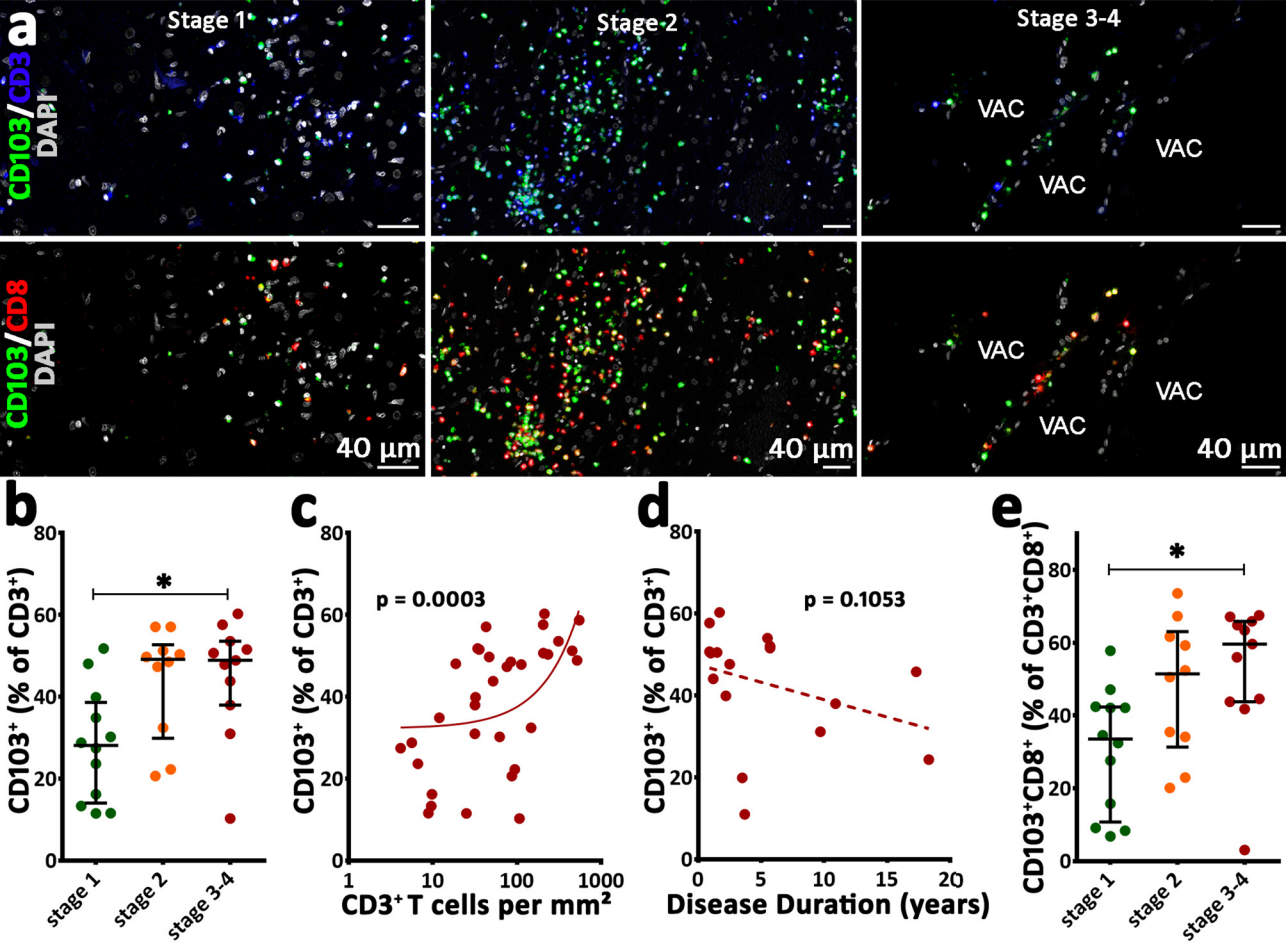
T_RM_ infiltration in the various stages of RE. Images originate from multiplex stainings for CD3, CD8, CD103, and DAPI. (**a**) Upper images show the stainings for CD103 and CD3 at the different stages, whereas lower images depict the stainings for CD103 in combination with CD8. In all stages, CD103^+^CD3^+^ T cells (T_RM_) are present, but these cells increase in stage 2 and stage 3–4. (**b**) The proportion of T_RM_ as a percentage of all CD3^+^ T cells. The proportion of T_RM_ cells significantly increases from stage 1 to stage 3–4. (**c**) The percentage of CD3^+^ T cells expressing CD103 positively correlates with the amount of infiltrating T cells (Spearman correlation *p* = 0.0003). (**d**) Shows that there is no correlation between the percentage of T_RM_/CD3^+^ T cells and the disease duration (Spearman correlation *p* = 0.1053). (**e**) The proportion of CD103 within the CD8^+^CD3^+^ T cell population. Again, a stage-dependent increase in the T_RM_ phenotype within the CD8^+^ T cell population from stage 1 to stage 3–4 was observed. Data are presented with a median with interquartile range; VAC = vacuole; (**b**, **e**): Kruskal-Wallis test was performed, asterisks represent the result of post hoc multiple comparisons (Dunn’s test): *p ≤ 0.05; (**c**, **d**): Spearman rank correlation was performed

T cells showing a T_RM_ phenotype were identified by staining for CD103 (from here on we refer to CD103^+^CD3^+^ T cells as T_RM_ unless otherwise specified). T_RM_ were present in the parenchyma in all examined cases and all stages (Fig. 2a**)**, with numbers spanning from 1.0 to 323.7 cells/mm² (median 18.8 cells/mm²) A significantly higher T_RM_ density was observed in stage 2 and stage 3–4 as compared to stage 1 **(**Fig. 2a, Supplementary Fig. 1b). Interestingly, also the proportion of T_RM_ increased significantly from stage 1 with a median of 28.11% of all CD3^+^ T cells to stage 3–4 (median 48.89% of all CD3^+^ T cells) (Fig. 2b). Furthermore, the ratio of T_RM_ positively correlated with the number of all infiltrating CD3^+^ T cells (Fig. 2c). However, the proportion of these infiltrating T_RM_ did not significantly vary with disease duration (Fig. 2d).

CD8^+^CD3^+^ T cells with a T_RM_ phenotype (CD103^+^CD8^+^CD3^+^ T cells) ranged from 0.1 to 123.7 cells/mm² and comprised a median of 43.75% of all CD8^+^CD3^+^ T cells. Again, a significant stage-dependent increase was observed from stage 1 to stage 3–4: within the CD8^+^ T cell population, a median of 33.48% in stage 1, 51.45% in stage 2, and 59.60% in stage 3–4 showed this T_RM_ phenotype (Fig. 2a and e, Supplementary Fig. 1c). The expression of CD103, which is not a faithful marker for CD4^+^ T_RM_ [17, 36, 37], within the CD4^+^CD3^+^ T cell population comprised a median of 0.66% (accounting for a median of 0.58% of all CD103^+^ T cells).

Another classical marker for T_RM_ is CD69. To confirm the presence of CD69^+^ T_RM_, we stained a number of cases for CD69. The proportion of CD69^+^ among all CD3^+^ T cells increased significantly from stage 1 (median = 5.14%) to stage 2 (median = 23.37%). The proportion of CD3^+^ T cells expressing both CD103 and CD69 significantly increased in stage 2 (median = 21.55%) compared to stage 1 (median = 4.69%). (Supplementary Fig. 2a-b). Furthermore, we performed staining for a third T_RM_ marker CD49a. Endothelial cells of blood vessels showed strong staining for CD49a. In contrast, expression on T cells was weak and much less frequent than CD103 and CD69, but generally co-expressed with these other T_RM_ markers. (Supplementary Fig. 2c and d).

### T cells likely acquire a T_RM_ phenotype in the parenchyma

**Fig. 3:**
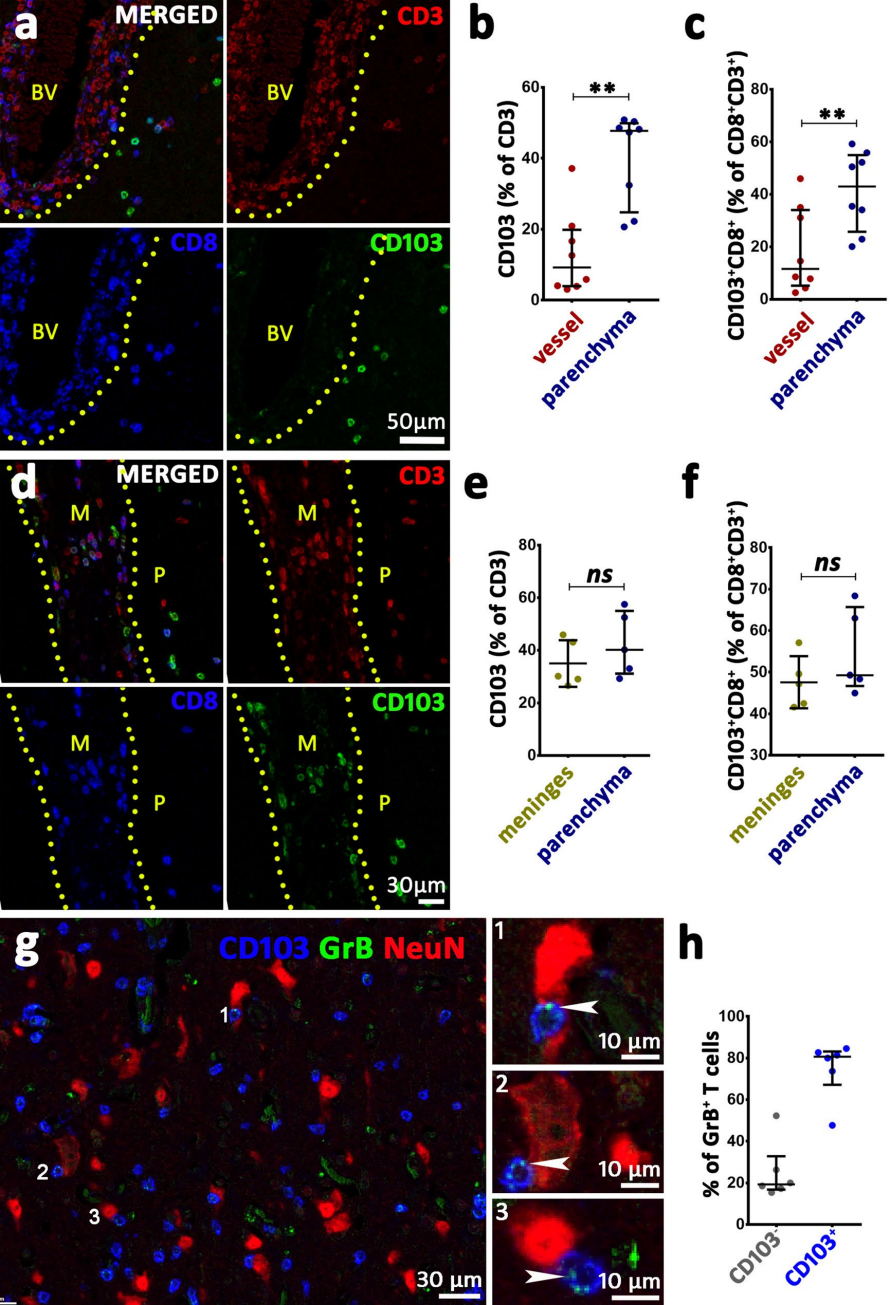
T_RM_ distribution in perivascular regions and brain parenchyma in RE cortical areas. (**a**) Multiplex immunofluorescence stainings. Shown are CD3^+^ T cells (red), CD8^+^ T cells (blue), and CD103^+^ T cells (green), whereas the merged panel shows colocalization of these markers in blood vessels (BV) and surrounding parenchyma of a stage 2 lesion. Notice that CD103 is almost exclusively found in the parenchyma. (**b**) Quantification of CD103^+^CD3^+^ T cells (T_RM_) as a percentage of all CD3^+^ T cells in the perivascular space versus the parenchyma. The proportion of T_RM_ is significantly higher in the parenchyma. (**c**) Quantification of CD8^+^ T_RM_ as a percentage of all CD8^+^CD3^+^ T cells in the perivascular space versus the parenchyma. The proportion of CD8^+^ T_RM_ is significantly higher in the parenchyma. (**d**) Representative multiplex immunofluorescence image of T_RM_ distribution in meninges and the brain parenchyma of a stage 2 lesion. CD3^+^ T cells (red), CD8^+^ T cells (blue), and CD103^+^ cells (green) are shown. The merged panel shows the colocalization of these cells. Here, T_RM_ are present in both the parenchyma (P) as well as in the meninges (M). (**e**) Quantification of T_RM_ as a percentage of all CD3^+^ T cells in the meninges versus the parenchyma. There is no statistical difference between these compartments. (**f**) Quantification of CD8^+^ T_RM_ as a percentage of all CD8^+^CD3^+^ T cells in the meninges versus the parenchyma. There is no statistical difference between these compartments. (**g**) Multiplex labeling for CD103 (blue), Granzyme-B (GrB) (green), and NeuN (red) in a stage 2 lesion. Inserts 1–3 indicate GrB^+^CD103^+^ cells in close apposition to neurons. GrB^+^ granules are indicated by white arrowheads. **(h)** The presence of GrB in CD103 positive and CD103 negative T cells. Data are presented as median with interquartile range; (**b**-**c**) and (**e**-**f**): Wilcoxon matched-pairs signed rank test was performed, asterisks represent significances **p ≤ 0.01

Most perivascular cuffs with CD3^+^ T cells were found in stage 2 areas. The median proportion of T_RM_ in these cuffs was 9.25%. In contrast, a median of 47.76% of T cells in the parenchyma were T_RM_ (Fig. 3a, b). A substantial proportion of the CD3^+^ T cells in the perivascular cuffs also stained positive for CD8. Likewise, the proportion of CD8^+^ T_RM_ (CD103^+^CD8^+^CD3^+^) increased in the parenchyma as compared to the perivascular space (42.99% in the parenchyma vs. 11.58% in the vascular space) (Fig. 3a, c). As for the CD69^+^ T cells, we could observe a similar pattern as with CD103 (Supplementary Fig. 2d). In addition, we analyzed tissue from five patients containing meninges and compared the proportion of T_RM_ in the meninges to those in the parenchyma. Surprisingly, the proportion of T_RM_ was similar in both compartments, as was the proportion of CD8^+^ T_RM_ (Fig. 3d-f). Further, we were interested in investigating whether T_RM_ showed effector cell function and could be actively involved in neuronal loss. To this end, we could observe T_RM_ expressing Granzyme-B (GrB) cytotoxic granules in close contact with neurons in all analyzed cases (Fig. 3g). Quantification of six samples revealed that a median of 80.7% of GrB^+^CD3^+^ T cells express CD103^+^ (Fig. 3h).

### T cell infiltration in the parenchyma, from activation to exhaustion

To further enlighten the dynamics of T cells in RE, we analyzed exhaustion-associated molecules Programmed Death 1 (PD-1), Cytotoxic T Lymphocyte Associated Protein 4 (CTLA-4), and Lymphocyte Activation Gene 3 (LAG-3) in the various stages of RE. Early experiments showed that the sensitivity of the used anti-CTLA4 antibody on our material seemed very low (only a few T cells in the parenchyma showed reactivity for CTLA-4). We thus refrained from performing further experiments with this antibody and concentrated on PD-1 and LAG-3.

### PD-1, but not LAG-3 expression, reflects chronic antigen stimulation

**Fig. 4:**
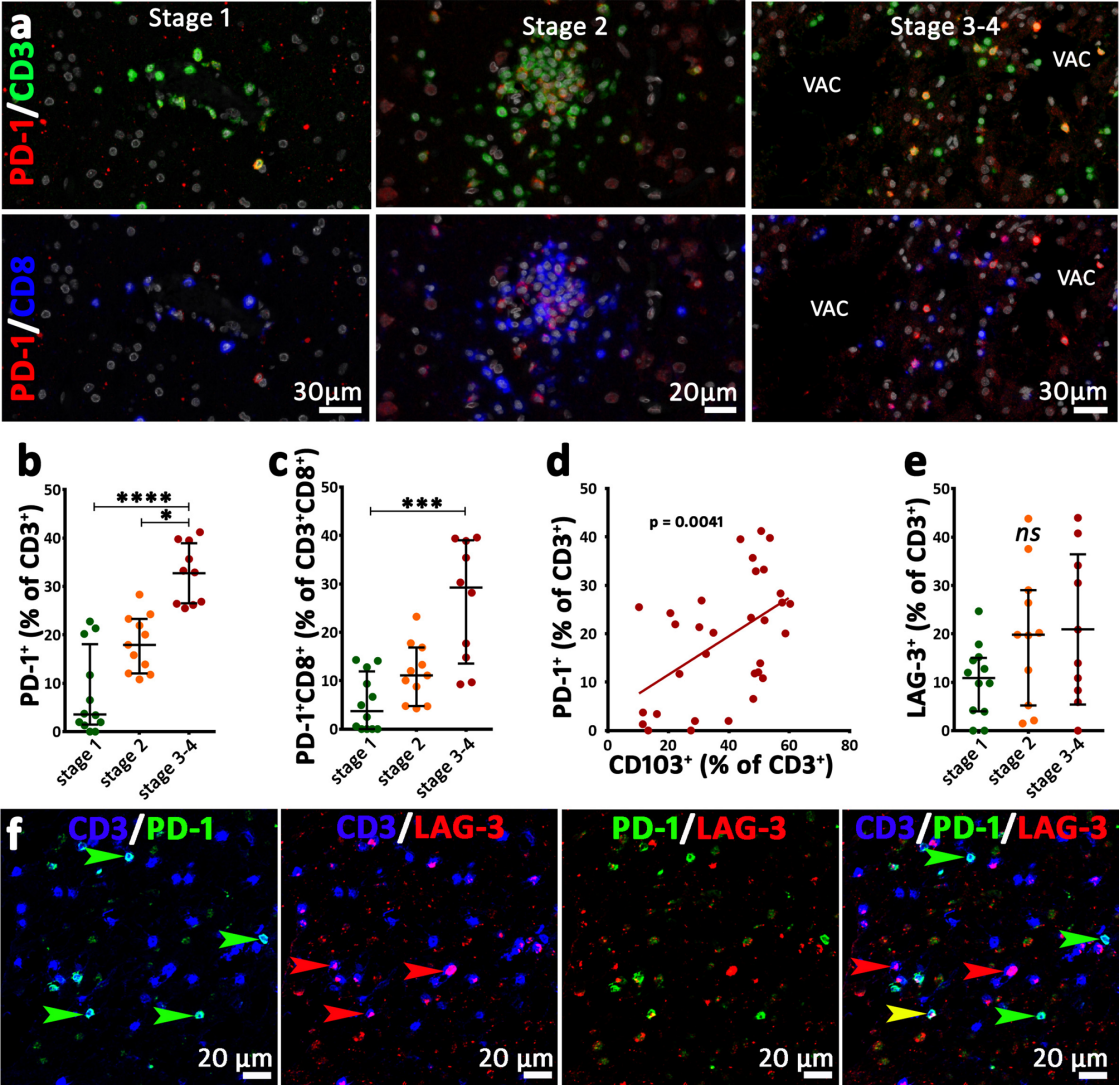
Exhaustion markers PD-1 and LAG-3 in RE brain **(a)** Images from multiplex stainings for CD3 (green), CD8 (blue), and PD-1 (red) in cortical sections from stage 1, stage 2, and stage 3–4 of RE. PD-1^+^ T cells are more prominent in stage 2 and stage 3–4 lesions than in stage 1 lesion. VAC indicates vacuoles in the stage 3–4 lesions. (**b**) The proportion of CD3^+^ T cells expressing PD-1 significantly increases from stage 1 to stage 3–4, as well as from stage 2 to stage 3–4. (**c**) Similarly, the proportion of CD8^+^CD3^+^ T cells expressing PD-1 shows a significant increase in stage 3–4. (**d**) Positive correlation of the proportion between CD103^+^CD3^+^ T cells (T_RM_) and PD-1^+^CD3^+^ T cells of all T cells (Spearman rank correlation *p* = 0.0041). (**e**) There is no significant change in the proportion of CD3^+^ T cells expressing LAG-3. (**f**) CD3^+^ T cells (blue) co-expressing PD-1 (green) and/or LAG-3 (red) in a stage 3–4 lesion. Many of the CD3^+^ T cells are either PD-1^+^ or LAG-3^+^. Only one cell, indicated by the yellow arrowhead in the merged CD3/PD-1/LAG-3 image, expresses both PD-1 and LAG-3. Data are presented as median with interquartile range; VAC = vacuole, (**b**, **c**, **e**): Kruskal Wallis test was performed, asterisks represent the result of post hoc multiple comparisons (Dunn’s test) ****p ≤ 0.0001, ***p ≤ 0.001, **p ≤ 0.01, *p ≤ 0.05; (**d**) Spearman rank correlation was performed

Our findings revealed PD-1 expression on CD3^+^ T cells across all stages (Fig. 4a), with cell densities ranging from 0 to 207.9 cells/mm². We observed an increase of absolute numbers of PD-1^+^CD3^+^ T cells from stage 1 to stage 2 and from stage 1 to stage 3–4 ( Supplementary Fig. 3a). Notably, the proportion of CD3^+^ T cells expressing PD-1 increased from stage 1 (median = 3.57%) and stage 2 (median = 17.94%) to stage 3–4 (median = 33.1%) (Fig. 4b). A similar trend was observed in CD3^+^CD8^+^ T cells, with significantly higher PD-1 expression on CD3^+^CD8^+^ T cells in stage 3–4 (median = 29.24%) compared to stage 1 (median 3.73%) (Fig. 4c). Furthermore, there was a positive correlation between the proportion of PD-1 and CD103 expression on T cells (Fig. 4d). CD3^+^ T cells expressing LAG-3 ranged from 0 to 391.3 cells/mm^2^ with a median of 5.6 cells/mm^2^. There was an increase in the density of LAG-3^+^CD3^+^ T cells in stage 2 and stage 3–4 compared to stage 1 (Supplementary Fig. 3b). In contrast to PD-1, we, however, noticed no stage-specific increase in the proportion of T cells expressing LAG-3. The numbers fluctuated between 0% and 43.98% (Fig. 4e). Similarly, CD3^+^CD8^+^ cytotoxic T cells expressing LAG-3 ranged from 0 to 53.85%, showing no stage-specific change (Supplementary Fig. 3c). Furthermore, we also analyzed the co-expression of LAG-3 and PD-1 on T cells. A median proportion of 1.96% (range: 0-20.4%) of all CD3^+^ T cells were positive for both markers (Fig. 4f, Supplementary Fig. 3d-e). Neither PD1^+^ T cells nor LAG-3^+^ T cells showed a significant change in proportion with ongoing disease duration (Supplementary Fig. 3f), which also accounts for the CD8^+^ T cell population (Supplementary Fig. 3g).

### The contribution of γδ^+^ T cells to neuronal loss

**Fig. 5:**
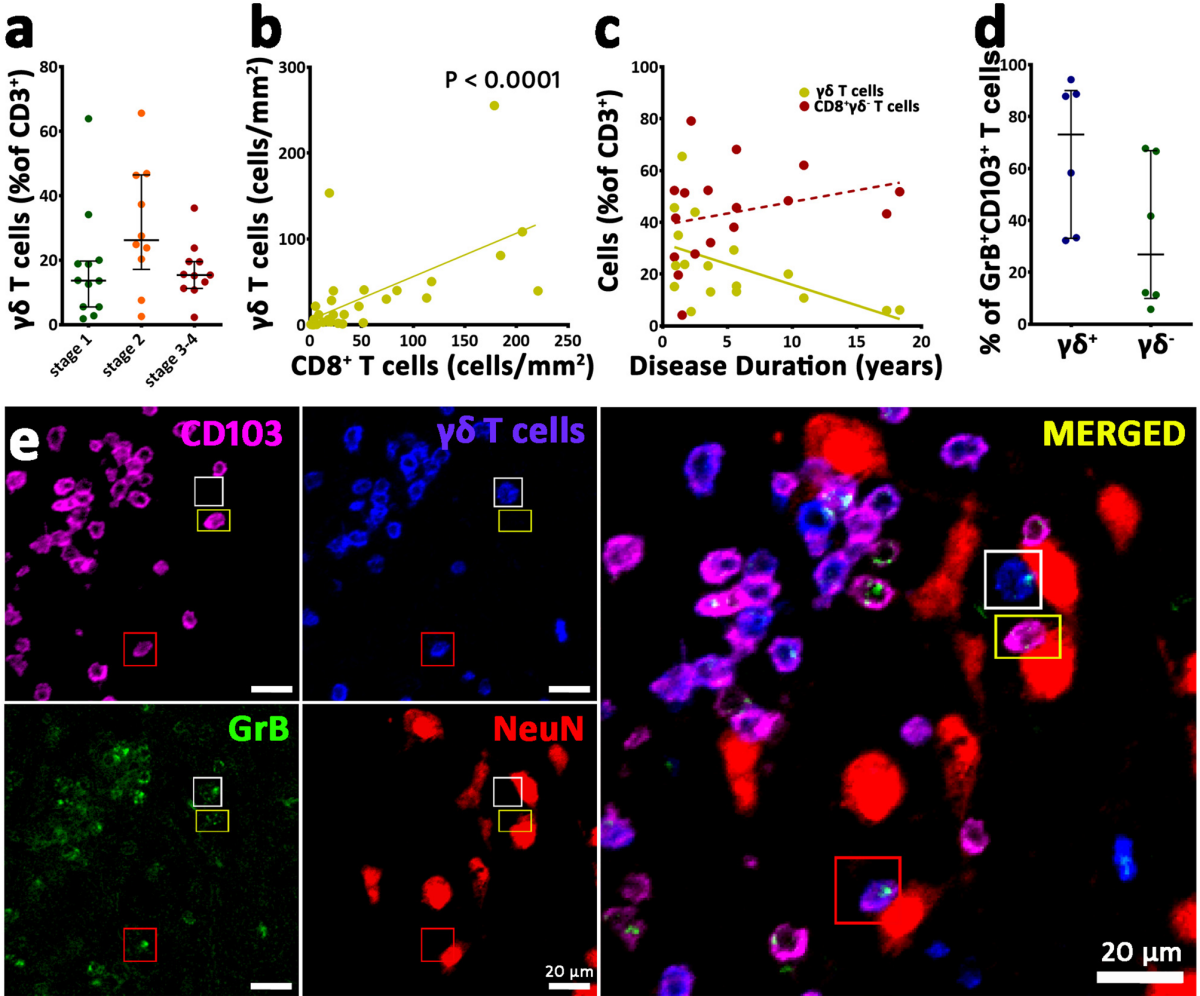
γδ+ T cells in Rasmussen encephalitis. (**a**) The proportion of CD3^+^ T cells expressing γδ TCR in the different stages of RE. This proportion did not significantly change across these disease stages. (**b**) A strong positive correlation, however, was observed between the number of γδ^+^CD3^+^ T cells and CD8^+^CD3^+^ T cells (*p* < 0.0001). (**c**) A negative correlation was identified between the proportion of infiltrating γδ^+^CD3^+^ T cells and disease duration (*p* = 0.0130). This correlation was not observed for the CD8^+^ (CD8^+^γδ^−^CD3^+^) T cells (*p* = 0.1473). (**d**) The proportion of γδ^+^ and γδ^−^ T cells of GrB^+^CD103^+^CD3^+^ T cells in six samples. No significant difference was observed between these groups. (**e**) Multiplex staining for CD103, δ TCR, GrB, and NeuN in a stage 2 lesion. This staining shows that γδ and CD103 expression can overlap with each other and with GrB. These cells can also be found in close proximity to NeuN^+^ neurons. The white rectangle indicates a CD103^−^GrB^+^γδ^+^ cell attached to a neuron. The yellow rectangle indicates a CD103^+^GrB^+^γδ^−^ cell attached to a neuron, and the red rectangle indicates a triple-positive CD103^+^GrB^+^γδ^+^ cell attached to a neuron. Data are presented as median with interquartile range; (**a**): Kruskal-Wallis test was performed; (**b-c**): Spearman rank correlation was performed; (**d**): Wilcoxon test was performed

Whereas most of the CD3^+^ T cells use the combined α- and β-T cell receptor (αβ-TCR), a smaller part of the T cells instead use a combination of γ- and δ-TCR. Previous studies [12, 13] showed that in RE, such γδ T cells are present. Here, we analyzed the presence and cytotoxic profile of these cells in more detail. First, infiltration of γδ^+^CD3^+^ T cells in the parenchyma reached a median density of 29.1 cells/mm^2^ in stage 2 and 31.3 cells/mm^2^ in stage 3–4 in comparison to a median number of 1.8 cells/mm^2^ to stage 1 (Supplementary Fig. 4a). The γδ^+^CD3^+^ T cells accounted for a median of 18.75% of all CD3^+^ T cells (Fig. 5a). In addition, within the γδ^+^CD3^+^ T cell population, a median proportion of 11.11% was also CD8^+^ while CD4^+^ expression of these γδ^+^CD3^+^ T cells was rare (median < 1%) (not shown). We did not observe a significant change in the proportion of γδ^+^CD3^+^ T cells within the CD3^+^ T cell population across different disease stages (Fig. 5a). A strong positive correlation however was found between the number of infiltrating γδ^+^CD3^+^ T cells and CD8^+^CD3^+^ T cells (Fig. 5b). Moreover, we identified a negative correlation between the proportion of infiltrating γδ^+^CD3^+^ T cells and the disease duration, while no such correlation was found for CD8^+^γδ^−^CD3^+^ T cells. (Fig. 5c).

We further determined the presence of CD103 on these γδ^+^CD3^+^ T cells. The majority (median = 71.51%, range = 33.3–100%) of all γδ^+^CD3^+^ T cells also stained positive for CD103 compared to a median of 43.75% in CD8^+^CD3^+^ T cells (Supplementary Fig. 4b). Unlike for CD8^+^CD3^+^ T cells, there was no significant increase of γδ^+^CD3^+^ T cells with T_RM_ phenotype between the stages (Supplementary Fig. 4b). Finally, the comparative analysis showed no difference in the proportion of CD103^+^γδ^+^CD3^+^ T cells within the parenchyma and within perivascular cuffs (Supplementary Fig. 4c). The question arises whether these γδ^+^ T cells have similar functions like the CD8^+^CD3^+^ cytotoxic T cells. We therefore performed multiplex staining for γδ^+^ TCR, CD3, CD103, and GrB, followed by quantitative analysis in six cases. A median of 73.10% (range = 32.26 − 94.28%) of all CD103^+^GrB^+^CD3^+^ T cells expressed the γδ T cell receptor (Fig. 5d). Like CD8^+^ T cells, γδ^+^ T cells not only expressed GrB but could also be found in close apposition to NeuN^+^ neurons, suggesting that also these γδ^+^ T cells are engaged in a cytotoxic T cell attack to neurons (Fig. 5e). In addition, by assessing the expression of proliferation markers (Ki67 and PCNA) we could observe that, like CD8^+^ T cells, γδ^+^ T cells are proliferating locally (Supplementary Fig. 4d,e). Finally, γδ^+^CD3^+^ T cells, with disease progression, showed increasing expression of PD-1; PD-1 expression on γδ^+^CD3^+^ T cells was significantly higher in stages 3–4 in comparison to stage 1. In contrast, LAG-3 expression on γδ^+^CD3^+^ T cells did not show an association with any disease stage (Supplementary Fig. 4f-i). Since we found that a considerable fraction of CD3^+^ T cells are γδ^+^ and express CD103, we repeated the studies of CD103^+^ cells, as shown in Supplementary Fig. 1b and Fig. 2b-d with the exclusion of γδ^+^CD3^+^ T cells; There was a significant increase in the densities of γδ^−^ T_RM_ from stage 1 to stage 2 and stages 3–4 (Supplementary Fig. 5a). Further, the increase in proportion of γδ^−^ T_RM_ among all γδ^−^CD3⁺ T cells from stage 1 to stages 3–4 remained (Supplementary Fig. 5b). Whereas the correlation between the proportion of γδ^−^ T_RM_ and the number of infiltrating γδ^−^CD3⁺ T cells persisted (Supplementary Fig. 5c), no correlation was observed between the proportion of γδ^−^ T_RM_ and disease duration (Supplementary Fig. 5d). Summarised, the presence of T_RM_ in different stages and throughout the disease course remained consistent, indicating that these findings are not depending on γδ^+^ T_RM_.

## Discussion

Our results show that during the disease course of RE, the number of T cells gradually decreases. This finding corroborates earlier findings showing that a decrease in T cells and microglial nodules during the disease is associated with a decrease in MRI abnormalities [38]. In GAD encephalitis, another CD8^+^ T cell-mediated neurodegenerative disease, we could observe the same phenomenon: early in the disease course, dense T cell infiltration is seen in the brain, whereas at later stages, T cells become gradually less, even though the assumed autoantigen (GAD65) is still present [39]. The early strong inflammation parallels an early neuronal loss. The same occurs in paraneoplastic CD8^+^ T cell-mediated diseases such as those associated with anti-Hu, anti-Ma2, or anti-Yo antibodies. In most anti-Hu and anti-Ma2 cases, CD8^+^ T cell infiltration is severe [32, 40–42] but in anti-Yo (paraneoplastic cerebellar degeneration) cases with more protracted disease duration, T cell numbers are much lower in the cerebellum or have even disappeared, leaving only the loss of cerebellar Purkinje cells and presence of microglial nodules as remnants of a CD8^+^ T cell-mediated attack [43, 44]. Overall, an early and severe neurodegeneration, together with decreasing numbers of cytotoxic CD8^+^ T cells in the CNS during the course of the disease, seems a likely scenario of CD8-mediated neurodegenerative diseases. Why CNS-infiltrating CD8^+^ T cells decrease over time remains unclear. In experimental models, γδ T cells have been shown to both regulate inflammation in the CNS and disease recovery via Fas/Fas ligand-induced apoptosis of encephalitogenic T cells [45]. Another possible explanation might be the compartmentalization of the immune response behind the blood-brain barrier (BBB) over time, as described in the chronic phase of the disease in MS [18].

Upon tissue entry, effector T cells use several mechanisms to establish residency, including the downregulation of molecules associated with tissue egress (e.g., CCR7, S1PR1, and S1PR5) and upregulation of molecules promoting tissue retention (e.g., CD69 and CD103) [46]. As shown in mouse models of virus infection, T_RM_ persist in the brain parenchyma after antigen clearance [47]. In RE, while the antigen remains enigmatic, it is suspected to be neuronal [8]. Here we showed that T_RM_ are present in the parenchyma and significantly less in the perivascular space. Furthermore, we demonstrated that the proportion of T_RM_ increase in end-stage (stage 3–4) lesions with severe neuronal loss. The increase in T_RM_ in the parenchyma may be explained by a specific enhanced migration of these cells from the perivascular space to the parenchyma. We, however, favor the probability that T_RM_ are induced locally in the CNS parenchyma and gradually increase in numbers due to neuronal loss and supposed antigen clearance. Although precursors destined for the T_RM_ fate have been identified in secondary lymphoid organs [48, 49], in most studies, the final differentiation into the T_RM_ program is suggested to occur after local antigen recognition following the migration of effector T cells in the tissues [50, 51].

Importantly, we could show that T_RM_ contained GrB^+^ granules and attacked neurons. This reveals that these T_RM_ can be present as effector cells and can be seen as essential drivers of human CNS autoimmunity, as illustrated in our CD8-mediated mouse models [19]. Unlike in the perivascular space, there was no difference in the number of T_RM_ between the parenchyma and the meninges. The clonal relationship between meningeal, perivascular, and parenchymal CD8^+^ T cells, harboring or not a T_RM_ phenotype, is still largely unknown. Analysis at the single cell level of TCR alpha and beta chain sequence of CD8^+^ T cells and CD8^+^ T_RM_ originating from these 3 anatomical compartments could allow a better understanding of their progenitor-progeny relationship and their migration within the CNS. In that respect, a recent study in human neurodegenerative diseases revealed that CD8^+^ T_RM_ can populate the leptomeninges, while TCR repertoire overlap between paired meningeal and parenchymal T cell samples suggests traffic between these two locations [52]. This is in line with our finding that the proportion of T_RM_ does not differ between the parenchyma and the meninges. Furthermore, it has been suggested that T_RM_ are organized in lymphoid niches near barriers in order to act as sentinels against possible reinfections [53]. Thus, our findings here could suggest that the meninges provide a protective niche in RE, ensuring T_RM_ maintenance [53] and a quick response against recurring perturbation.

In oncology, immune checkpoint inhibitors (ICIs) are a revolutionary therapeutic strategy. Currently, anti-PD-1, anti-CTLA-4, as well as anti-LAG-3 antibodies are approved [54, 55]. One common side effect of ICIs is the induction of autoimmune-like inflammation throughout the body, including encephalitis [56, 57]. Cytotoxic T cells are likely to be the driving force [31]. Tissue-resident memory T cells (T_RM_) have gained attention for their role in enhancing the efficacy of ICIs [58], and recent findings suggest that T_RM_ might predict the efficacy of ICIs and influence it positively [59]. In tumors with chronic antigen stimulation, the co-expression of CD103 and PD-1 on T cells was reported [58, 60]. Moreover, in lung cancer, it has been shown that the density of CD8^+^ T_RM_ correlates with a positive response to anti-PD-1 antibody treatment. Moreover, this cell population increases in most patients, which might reflect a reversible exhausted state [61]. Here, we observed a positive correlation between the infiltration of CD103^+^ T cells and PD-1 expression. CD103 expression remained constant between stage 2 and stage 3–4. PD-1, in contrast, showed an increase between these stages. While PD-1 expression is widely associated with antigen exposure and T cell modulation [62, 63], this observation suggests that T_RM_ in RE may shift in their functional state towards a self-regulating process. Further studies are necessary to evaluate the exact role of PD-1 expression on T cells in this setting. LAG-3 is the most recent clinically approved checkpoint inhibitor. It, among others, interacts with MHC class II molecules to regulate T cell activation. Synergistic effects of the blockage of both PD-1 and LAG-3 have been reported [64]. As seen in supplementary Fig. 3e, LAG-3 and PD-1 populations show little overlap. Further, in contrast to the stage-dependent increase of PD-1, we did not see an increase of LAG-3. While it was shown that the inhibition of both LAG-3 and PD-1 has synergistic anti-tumor effects, their mechanism of action have been suggested to differ [64]. Although the cause of RE is unclear, our findings here also suggest that the actions of PD-1 and LAG-3 positive cells differ and that LAG-3, in contrast to PD-1, may be involved in an antigen-independent intrinsic immune regulatory process.

Unlike the more common αβ T cells, γδ T cells can recognize microbial molecules, phosphoantigens, or stress-related molecules [65–67] in an MHC-independent way [68]. We confirm the previously described presence of γδ^+^ T cells in RE [12, 13]. More importantly, we analyzed the contribution of those cells to neuronal loss and immune regulation in more detail. Previously, we have shown that in microglial nodules in RE, Toll-like receptors 4 and 7 are upregulated. In addition, we recognized an increase in DNA-binding proteins like high-mobility group box 1 (HMGB1) [7]. These findings suggested a neuronal reaction to a possible infectious agent. Since γδ^+^ T cells recognize and react to stress-induced antigens (such as HMGB1), our results suggest that in RE the γδ^+^ T cells, by their anti-stress actions, possibly by local communication with microglia within the microglial nodules, may broaden and enhance the inflammatory response in a way, which is independent from the initial disease-driving antigen. We propose that through this mechanism, γδ^+^ T cells may complement the αβ T cell response by amplifying tissue damage beyond antigen-specific cytotoxicity.

Interestingly, γδ^+^ T cells were more prevalent in cases with shorter disease duration. That this especially occurs in the early stage of the disease may suggest that these γδ^+^ T cells represent one of the means to increase the efficacy of the immune response as fast as possible. Moreover, it has been described that γδ^+^ T cells can promote epilepsy by producing proinflammatory cytokines (IL-17, GM-CSF) that enhance neuronal excitability and contribute to seizure severity [69]. So far, only CD8^+^CD3^+^ T cells have been reported to be responsible for neuronal loss [8]. Here, we reveal that GrB-expressing γδ^+^ T cells are found attached to neurons and thus contribute to neuronal destruction during RE. Theoretically, the TCR repertoire of γδ^+^ T cells exceeds that of αβ^+^ Τ cells, but γδ^+^ T cells are still assumed as more invariant T cells that only use a restricted set of γδ TCRs [70]. This may be advantageous while investigating the antigen-specificity of these cells. Further, our results show that these γδ^+^ T cells can express CD103. Notably, in this study, the fraction of γδ^+^ T cells expressing T_RM_ features was significantly higher than that of αβ^+^ Τ cells. Since the γδ^+^ T cells, in contrast to CD8^+^ T cells, already expressed CD103 within the perivascular space rather than after migration into the parenchyma, and since the majority of these γδ^+^ T cells don´t co-express CD8, the question arises whether these cells can be regarded as faithful T_RM_.

## Conclusion

Our study provides new and important insights into the mechanisms of brain inflammation and neuronal injury in RE. It indicates that the initial lesions are triggered by MHC Class I restricted CD8^+^ T-lymphocytes, which recognize an antigen presented by neurons and which induce an immune-mediated neuronal injury. As in other diseases of CD8^+^ T-cell mediated inflammation in the CNS, a proportion of the pathogenic T-cells become trapped within the CNS and differentiate into tissue resident memory cells. These cells remain as guardians within the parenchyma or the meninges in an inactive or exhausted state, but may become reactivated upon re-appearance of the specific target antigen and propagate low-grade chronic inflammation. However, acute and chronic disease lesions in RE are further propagated by an additional T-cell population with γδ T-cell receptors. These γδ T-cells are the dominant cells at the onset of disease. Moreover, these cells are tissue resident effector T-cells and show temporally and focally restricted reactivation as reflected by their proliferation. Since such cells recognize a restricted number of antigens, it may become easier to define their RE-specific immune responses and to target them through desensitization approaches.

## Electronic supplementary material

Below is the link to the electronic supplementary material.

Supplementary Material 1

Supplementary Material 2

Supplementary Material 3

Supplementary Material 4

Supplementary Material 5

Supplementary Material 6

Supplementary Material 7

Supplementary Material 8

## Data Availability

All data generated or analysed during this study are included in this published article (and its supplementary information files). The raw counting data are available from the corresponding author upon reasonable request.

## Abbreviations

RE: Rasmussen Encephalitis
GrB: Granzyme-B
PBMCs: Peripheral Blood Mononuclear Cells
NSG mouse: NOD scid gamma mouse (immunodeficient mouse model)
MHC: Major Histocompatibility Complex
T_RM_: Tissue-Resident Memory T cells
PD-1: Programmed Death-1
CTLA-4: Cytotoxic T-Lymphocyte Associated Protein 4
LAG-3: Lymphocyte Activation Gene-3
FFPE: Formalin-Fixed Paraffin-Embedded
Iba1: Ionized Calcium Binding Adapter Molecule 1
HLA-DR: Human Leukocyte Antigen - DR isotype
GFAP: Glial Fibrillary Acidic Protein
NeuN: Neuronal Nuclei
HIER: Heat-Induced Epitope Retrieval
CSA: Catalyzed Signal Amplification
DAPI: 4′,6-diamidino-2-phenylindole (a fluorescent stain for nuclei)
AR9: Antigen Retrieval Buffer pH 9.0
HRP: Horseradish Peroxidase
MRI: Magnetic Resonance Imaging
GAD: Glutamic Acid Decarboxylase
MS: Multiple Sclerosis
BBB: Blood-Brain Barrier
CCR7: C-C Chemokine Receptor Type 7
S1PR1: Sphingosine-1-Phosphate Receptor 1

## References

[1] Aguilar MJ, Rasmussen T. Role of encephalitis in pathogenesis of epilepsy. Arch Neurol. 1960;2(6):663–76. 13792099 10.1001/archneur.1960.03840120069008

[2] Vining EPG, Freeman JM, Brandt J, Carson BS, Uematsu S. Progressive unilateral encephalopathy of childhood (Rasmussen’s Syndrome): A Reappraisal. Epilepsia. 1993;34(4):639–50. 8330574 10.1111/j.1528-1157.1993.tb00441.x

[3] Wiendl H, Gross CC, Bauer J, Merkler D, Prat A, Liblau R. Fundamental mechanistic insights from rare but paradigmatic neuroimmunological diseases. Nat Rev Neurol. 2021;17(7):433–47. 34050331 10.1038/s41582-021-00496-7

[4] Pardo CA, Vining EPG, Guo L, Skolasky RL, Carson BS, Freeman JM. The pathology of rasmussen syndrome: stages of cortical involvement and neuropathological studies in 45 hemispherectomies. Epilepsia. 2004;45(5):516–26. 15101833 10.1111/j.0013-9580.2004.33103.x

[5] Varadkar S, Bien CG, Kruse CA, Jensen FE, Bauer J, Pardo CA, et al. Rasmussen’s encephalitis: clinical features, pathobiology, and treatment advances. Lancet Neurol. 2014;13(2):195–205. 24457189 10.1016/S1474-4422(13)70260-6 PMC4005780

[6] Rasmussen T, Olszewski J, Lloyd-Smith D. Focal seizures due to chronic localized encephalitis. Neurology. 1958;8(6):435–435. 13566382 10.1212/wnl.8.6.435

[7] Tröscher AR, Wimmer I, Quemada-Garrido L, Köck U, Gessl D, Verberk SGS, et al. Microglial nodules provide the environment for pathogenic T cells in human encephalitis. Acta Neuropathol (Berl). 2019;137(4):619–35. 30663001 10.1007/s00401-019-01958-5 PMC6426829

[8] Bien CG, Bauer J, Deckwerth TL, Wiendl H, Deckert M, Wiestler OD, et al. Destruction of neurons by cytotoxic T cells: A new pathogenic mechanism in rasmussen’s encephalitis. Ann Neurol. 2002;51(3):311–8. 11891826 10.1002/ana.10100

[9] Schwab N, Bien CG, Waschbisch A, Becker A, Vince GH, Dornmair K, et al. CD8 + T-cell clones dominate brain infiltrates in rasmussen encephalitis and persist in the periphery. Brain. 2009;132(5):1236–46. 19179379 10.1093/brain/awp003

[10] Kebir H, Carmant L, Fontaine F, Béland K, Bosoi CM, Sanon NT, et al. Humanized mouse model of rasmussen’s encephalitis supports the immune-mediated hypothesis. J Clin Invest. 2018;128(5):2000–9. 29629902 10.1172/JCI97098 PMC5919802

[11] Schneider-Hohendorf T, Mohan H, Bien CG, Breuer J, Becker A, Görlich D, et al. CD8 + T-cell pathogenicity in rasmussen encephalitis elucidated by large-scale T-cell receptor sequencing. Nat Commun. 2016;7(1):11153. 27040081 10.1038/ncomms11153 PMC4822013

[12] Owens GC, Erickson KL, Malone CC, Pan C, Huynh MN, Chang JW, et al. Evidence for the involvement of gamma delta T cells in the immune response in rasmussen encephalitis. J Neuroinflammation. 2015;12(1):134. 26186920 10.1186/s12974-015-0352-2 PMC4506578

[13] Al Nimer F, Jelcic I, Kempf C, Pieper T, Budka H, Sospedra M, et al. Phenotypic and functional complexity of brain-infiltrating T cells in rasmussen encephalitis. Neurol Neuroimmunol Neuroinflammation. 2018;5(1):e419. 10.1212/NXI.0000000000000419 PMC5733246 29259996

[14] Chien Yhsiu, Meyer C, Bonneville M, γδ T, Cells. First line of defense and beyond. Annu Rev Immunol. 2014;32(1):121–55. 24387714 10.1146/annurev-immunol-032713-120216

[15] Zeng X, Meyer C, Huang J, Newell EW, Kidd BA, Wei YL, et al. Gamma delta T cells recognize haptens and mount a hapten-specific response. eLife. 2014;3:e03609. 25255099 10.7554/eLife.03609 PMC4174581

[16] Kimura K, Nishigori R, Hamatani M, Sawamura M, Ashida S, Fujii C, et al. Resident Memory-like CD8 + T cells are involved in chronic inflammatory and neurodegenerative diseases in the CNS. Neurol Neuroimmunol Neuroinflammation. 2024;11(1):e200172. 10.1212/NXI.0000000000200172 PMC10691221 37949669

[17] Smolders J, Heutinck KM, Fransen NL, Remmerswaal EBM, Hombrink P, Ten Berge IJM, et al. Tissue-resident memory T cells populate the human brain. Nat Commun. 2018;9(1):4593. 30389931 10.1038/s41467-018-07053-9 PMC6214977

[18] Machado-Santos J, Saji E, Tröscher AR, Paunovic M, Liblau R, Gabriely G, et al. The compartmentalized inflammatory response in the multiple sclerosis brain is composed of tissue-resident CD8 + T lymphocytes and B cells. Brain. 2018;141(7):2066–82. 29873694 10.1093/brain/awy151 PMC6022681

[19] Frieser D, Pignata A, Khajavi L, Shlesinger D, Gonzalez-Fierro C, Nguyen XH, et al. Tissue-resident CD8 + T cells drive compartmentalized and chronic autoimmune damage against CNS neurons. Sci Transl Med. 2022;14(640):eabl6157. 35417189 10.1126/scitranslmed.abl6157

[20] Wherry EJ. T cell exhaustion. Nat Immunol. 2011;12(6):492–9. 21739672 10.1038/ni.2035

[21] Blackburn SD, Shin H, Haining WN, Zou T, Workman CJ, Polley A, et al. Coregulation of CD8 + T cell exhaustion by multiple inhibitory receptors during chronic viral infection. Nat Immunol. 2009;10(1):29–37. 19043418 10.1038/ni.1679 PMC2605166

[22] Grosso JF, Goldberg MV, Getnet D, Bruno TC, Yen HR, Pyle KJ, et al. Functionally distinct LAG-3 and PD-1 subsets on activated and chronically stimulated CD8 T cells. J Immunol. 2009;182(11):6659–69. 19454660 10.4049/jimmunol.0804211 PMC3082361

[23] Byrne A, Savas P, Sant S, Li R, Virassamy B, Luen SJ, et al. Tissue-resident memory T cells in breast cancer control and immunotherapy responses. Nat Rev Clin Oncol. 2020;17(6):341–8. 32112054 10.1038/s41571-020-0333-y

[24] Vogrig A, Fouret M, Joubert B, Picard G, Rogemond V, Pinto AL, et al. Increased frequency of anti-Ma2 encephalitis associated with immune checkpoint inhibitors. Neurol Neuroimmunol Neuroinflammation. 2019;6(6):e604. 10.1212/NXI.0000000000000604 PMC6705619 31454760

[25] Hashimoto R, Tanabe E, Otsuka Y, Yoneda Y, Kageyama Y. Anti-Ma2-Associated limbic encephalitis after termination of immune checkpoint inhibitor therapy for malignant pleural mesothelioma. Case Rep Neurol. 2021;13(3):724–8. 34950011 10.1159/000519763 PMC8647130

[26] Farina A, Villagrán-García M, Ciano-Petersen NL, Vogrig A, Muñiz-Castrillo S, Taillandier L, et al. Anti-Hu antibodies in patients with neurologic side effects of immune checkpoint inhibitors. Neurol Neuroimmunol Neuroinflammation. 2023;10(1):e200058. 10.1212/NXI.0000000000200058 PMC9709718 36446613

[27] Farina A, Villagrán-García M, Vogrig A, Zekeridou A, Muñiz-Castrillo S, Velasco R, et al. Neurological adverse events of immune checkpoint inhibitors and the development of paraneoplastic neurological syndromes. Lancet Neurol. 2024;23(1):81–94. 38101905 10.1016/S1474-4422(23)00369-1

[28] Segal Y, Soltys J, Clarkson BDS, Howe CL, Irani SR, Pittock SJ. Toward curing neurological autoimmune disorders: biomarkers, immunological mechanisms, and therapeutic targets. Neuron. 2025;113(3):345–79. 39809275 10.1016/j.neuron.2024.12.006 PMC13102114

[29] Fonseca E, Cabrera-Maqueda JM, Ruiz-García R, Naranjo L, Diaz-Pedroche C, Velasco R, et al. Neurological adverse events related to immune-checkpoint inhibitors in spain: a retrospective cohort study. Lancet Neurol. 2023;22(12):1150–9. 37977714 10.1016/S1474-4422(23)00335-6

[30] Rosenfeld MR, Titulaer MJ, Dalmau J. Paraneoplastic syndromes and autoimmune encephalitis: five new things. Neurol Clin Pract. 2012;2(3):215–23. 23634368 10.1212/CPJ.0b013e31826af23e PMC3613202

[31] Yshii LM, Gebauer CM, Pignolet B, Mauré E, Quériault C, Pierau M, et al. CTLA4 blockade elicits paraneoplastic neurological disease in a mouse model. Brain. 2016;139(11):2923–34. 27604307 10.1093/brain/aww225

[32] Barnett M. Paraneoplastic brain stem encephalitis in a woman with anti-Ma2 antibody. J Neurol Neurosurg Psychiatry. 2001;70(2):222–5. 11160472 10.1136/jnnp.70.2.222 PMC1737194

[33] Bien CG. Pathogenesis, diagnosis and treatment of rasmussen encephalitis: A european consensus statement. Brain. 2005;128(3):454–71. 15689357 10.1093/brain/awh415

[34] Bauer J, Lassmann H. Neuropathological Techniques to Investigate Central Nervous System Sections in Multiple Sclerosis. In: Weissert R, editor. Multiple Sclerosis [Internet]. New York, NY: Springer New York; 2014 [cited 2024 Sep 18]. pp. 211–29. (Methods in Molecular Biology; vol. 1304). Available from: http://link.springer.com/10.1007/7651_2014_151 10.1007/7651_2014_151 25520281

[35] Bankhead P, Loughrey MB, Fernández JA, Dombrowski Y, McArt DG, Dunne PD, et al. QuPath: open source software for digital pathology image analysis. Sci Rep. 2017;7(1):16878. 29203879 10.1038/s41598-017-17204-5 PMC5715110

[36] Klicznik MM, Morawski PA, Höllbacher B, Varkhande SR, Motley SJ, Kuri-Cervantes L, et al. Human CD4 + CD103 + cutaneous resident memory T cells are found in the circulation of healthy individuals. Sci Immunol. 2019;4(37):eaav8995. 31278120 10.1126/sciimmunol.aav8995 PMC7057121

[37] Li J, Xiao C, Li C, He J. Tissue-resident immune cells: from defining characteristics to roles in diseases. Signal Transduct Target Ther. 2025;10(1):12. 39820040 10.1038/s41392-024-02050-5 PMC11755756

[38] Bien CG, Urbach H, Deckert M, Schramm J, Wiestler OD, Lassmann H, et al. Diagnosis and staging of rasmussen’s encephalitis by serial MRI and histopathology. Neurology. 2002;58(2):250–7. 11805253 10.1212/wnl.58.2.250

[39] Tröscher AR, Mair KM, De Verdú L, Köck U, Steinmaurer A, Baier H, et al. Temporal lobe epilepsy with GAD antibodies: neurons killed by T cells not by complement membrane attack complex. Brain. 2023;146(4):1436–52. 36314080 10.1093/brain/awac404 PMC10115353

[40] Bernal F, Graus F, Pifarré À, Saiz A, Benyahia B, Ribalta T. Immunohistochemical analysis of anti-Hu-associated paraneoplastic encephalomyelitis. Acta Neuropathol (Berl). 2002;103(5):509–15. 11935268 10.1007/s00401-001-0498-0

[41] Blumenthal DT, Salzman KL, Digre KB, Jensen RL, Dunson WA, Dalmau J. Early pathologic findings and long-term improvement in anti-Ma2–associated encephalitis. Neurology. 2006;67(1):146–9. 16832096 10.1212/01.wnl.0000223647.83708.20

[42] Bien CG, Vincent A, Barnett MH, Becker AJ, Blumcke I, Graus F, et al. Immunopathology of autoantibody-associated encephalitides: clues for pathogenesis. Brain. 2012;135(5):1622–38. 22539258 10.1093/brain/aws082

[43] Storstein A, Krossnes BK, Vedeler CA. Morphological and immunohistochemical characterization of paraneoplastic cerebellar degeneration associated with yo antibodies. Acta Neurol Scand. 2009;120(1):64–7. 19486326 10.1111/j.1600-0404.2008.01138.x

[44] Verschuuren J, Chuang L, Rosenblum MK, Lieberman F, Pryor A, Posner JB, et al. Inflammatory infiltrates and complete absence of purkinje cells in anti-Yo-associated paraneoplastic cerebellar degeneration. Acta Neuropathol (Berl). 1996;91(5):519–25. 8740233 10.1007/s004010050460

[45] Ponomarev ED, Dittel BN. Gamma delta T cells regulate the extent and duration of inflammation in the central nervous system by a fas ligand-dependent mechanism. J Immunol Baltim Md 1950. 2005;174(8):4678–87. 10.4049/jimmunol.174.8.4678 15814692

[46] Osman M, Park SL, Mackay LK. Tissue-resident memory T (T_RM_) cells: Front-line workers of the immune system. Eur J Immunol. 2023;53(11):e2250060. 36597841 10.1002/eji.202250060

[47] Wakim LM, Woodward-Davis A, Bevan MJ. Memory T cells persisting within the brain after local infection show functional adaptations to their tissue of residence. Proc Natl Acad Sci U S A. 2010;107(42):17872–9. 20923878 10.1073/pnas.1010201107 PMC2964240

[48] Kok L, Dijkgraaf FE, Urbanus J, Bresser K, Vredevoogd DW, Cardoso RF, et al. A committed tissue-resident memory T cell precursor within the circulating CD8 + effector T cell pool. J Exp Med. 2020;217(10):e20191711. 32728699 10.1084/jem.20191711 PMC7537386

[49] Parga-Vidal L, Behr FM, Kragten NAM, Nota B, Wesselink TH, Kavazović I, et al. Hobit identifies tissue-resident memory T cell precursors that are regulated by eomes. Sci Immunol. 2021;6(62):eabg3533. 34417257 10.1126/sciimmunol.abg3533

[50] Van Gisbergen KPJM, Zens KD, Münz C. T-cell memory in tissues. Eur J Immunol. 2021;51(6):1310–24. 33837521 10.1002/eji.202049062

[51] Christo SN, Park SL, Mueller SN, Mackay LK. The multifaceted role of Tissue-Resident memory T cells. Annu Rev Immunol. 2024;42(1):317–45. 38941605 10.1146/annurev-immunol-101320-020220

[52] Hobson R, Levy SHS, Flaherty D, Xiao H, Ciener B, Reddy H et al. Clonal CD8 T Cells Accumulate in the Leptomeninges and Communicate with Microglia in Human Neurodegeneration. Res Sq. 2024;rs.3.rs-3755733.

[53] Takamura S. Niches for the Long-Term maintenance of Tissue-Resident memory T cells. Front Immunol. 2018;9:1214. 29904388 10.3389/fimmu.2018.01214 PMC5990602

[54] Alturki NA. Review of the immune checkpoint inhibitors in the context of Cancer treatment. J Clin Med. 2023;12(13):4301. 37445336 10.3390/jcm12134301 PMC10342855

[55] FDA approves anti-LAG3 checkpoint. Nat Biotechnol. 2022;40(5):625–625. 35577946 10.1038/s41587-022-01331-0

[56] Yshii LM, Hohlfeld R, Liblau RS. Inflammatory CNS disease caused by immune checkpoint inhibitors: status and perspectives. Nat Rev Neurol. 2017;13(12):755–63. 29104289 10.1038/nrneurol.2017.144

[57] Cuzzubbo S, Javeri F, Tissier M, Roumi A, Barlog C, Doridam J, et al. Neurological adverse events associated with immune checkpoint inhibitors: review of the literature. Eur J Cancer. 2017;73:1–8. 28064139 10.1016/j.ejca.2016.12.001

[58] Webb JR, Milne K, Nelson BH. PD-1 and CD103 are widely coexpressed on prognostically favorable intraepithelial CD8 T cells in human ovarian Cancer. Cancer Immunol Res. 2015;3(8):926–35. 25957117 10.1158/2326-6066.CIR-14-0239

[59] Sato H, Meng S, Hara T, Tsuji Y, Arao Y, Sasaki K, et al. Tissue-Resident memory T cells in gastrointestinal cancers: prognostic significance and therapeutic implications. Biomedicines. 2024;12(6):1342. 38927549 10.3390/biomedicines12061342 PMC11202222

[60] Pearce H, Croft W, Nicol SM, Margielewska-Davies S, Powell R, Cornall R, et al. Tissue-Resident memory T cells in pancreatic ductal adenocarcinoma coexpress PD-1 and TIGIT and functional inhibition is reversible by dual antibody blockade. Cancer Immunol Res. 2023;11(4):435–49. 36689623 10.1158/2326-6066.CIR-22-0121 PMC10068448

[61] Corgnac S, Malenica I, Mezquita L, Auclin E, Voilin E, Kacher J, et al. CD103 + CD8 + T_RM_ cells accumulate in tumors of Anti-PD-1-Responder lung Cancer patients and are Tumor-Reactive lymphocytes enriched with Tc17. Cell Rep Med. 2020;1(7):100127. 33205076 10.1016/j.xcrm.2020.100127 PMC7659589

[62] Liu R, Li HF, Li S. PD-1-mediated inhibition of T cell activation: mechanisms and strategies for cancer combination immunotherapy. Cell Insight. 2024;3(2):100146. 38425643 10.1016/j.cellin.2024.100146 PMC10901852

[63] Bally APR, Austin JW, Boss JM. Genetic and epigenetic regulation of PD-1 expression. J Immunol. 2016;196(6):2431–7. 26945088 10.4049/jimmunol.1502643 PMC4780223

[64] Andrews LP, Butler SC, Cui J, Cillo AR, Cardello C, Liu C, et al. LAG-3 and PD-1 synergize on CD8 + T cells to drive T cell exhaustion and hinder autocrine IFN-γ-dependent anti-tumor immunity. Cell. 2024;187(16):4355–e437222. 39121848 10.1016/j.cell.2024.07.016 PMC11323044

[65] Hirsh MI, Junger WG. Roles of heat shock proteins and γδT cells in inflammation. Am J Respir Cell Mol Biol. 2008;39(5):509–13. 18566334 10.1165/rcmb.2008-0090TR PMC2574523

[66] Dong R, Zhang Y, Xiao H, Zeng X. Engineering γδ T cells: recognizing and activating on their own way. Front Immunol. 2022;13:889051. 35603176 10.3389/fimmu.2022.889051 PMC9120431

[67] Groh V, Steinle A, Bauer S, Spies T. Recognition of Stress-Induced MHC molecules by intestinal epithelial γδ T cells. Science. 1998;279(5357):1737–40. 9497295 10.1126/science.279.5357.1737

[68] Born WK, Kemal Aydintug M, O’Brien RL. Diversity of γδ T-cell antigens. Cell Mol Immunol. 2013;10(1):13–20. 23085946 10.1038/cmi.2012.45 PMC4003174

[69] Xu D, Robinson AP, Ishii T, Duncan DS, Alden TD, Goings GE, et al. Peripherally derived T regulatory and γδ T cells have opposing roles in the pathogenesis of intractable pediatric epilepsy. J Exp Med. 2018;215(4):1169–86. 29487082 10.1084/jem.20171285 PMC5881465

[70] Fichtner AS, Ravens S, Prinz I. Human γδ TCR repertoires in health and disease. Cells. 2020;9(4):800. 32225004 10.3390/cells9040800 PMC7226320

