# Supplementary material for: The dynamics of brain T cell populations during the course of rasmussen encephalitis: from expansion to exhaustion
Source: J Neuroinflammation. 2025 Jun 12;22:155. doi: 10.1186/s12974-025-03477-5 (PMC12164096; doi:10.1186/s12974-025-03477-5)
Supplement: Supplementary file 1 — Supplementary Material 1 [file 12974_2025_3477_MOESM1_ESM.docx]

****Supplementary Figure 1. CD4⁺ and CD8⁺ T cell proportions remain stable over disease duration, while T_RM_ cell density increases from stage 1 to 3–4****

**(a**) Percentage of CD8^+^CD3^+^ (red) and CD4^+^CD3^+^ T cells (blue) among all parenchymal T cells during the disease duration of RE. No increase or decrease was found (Spearman rank correlation p= 0.1930 and p = 0.4105, respectively). (**b**) The quantitative density of CD103^+^CD3^+^ T cells (T_RM_) across disease stages. Here we see a significant increase in T_RM_ from stage 1 to stage 2 and stage 3-4. (**c**) The density of CD8^+^ T_RM_ shows an increase from stage 1 to stage 2 and stage 3-4. (**a**) Spearman’s rank correlation was performed; (**b-c**) Kruskal-Wallis test was performed, asterisks represent the result of post-hoc multiple comparison (Dunn’s test) ***p ≤0.001, **p ≤0.01.

****Supplementary Figure. 2. Characterization of different T_RM_ markers in RE****

**(a) The proportion of CD69^+^CD3^+^ T cells among CD3^+^ T cells across the stages 1-4. A significant increase was observed from stage 1 (median = 5.14%) to stage 2 (median = 23.37 %). (b) Similarly, the ratio of CD103^+^CD69^+^CD3^+^ T cells among CD3^+^ T cells is significantly higher in stage 2 (median = 21.55%) compared to stage 1 (median = 4.69%). (c) Representative multiplex fluorescence staining of different markers for T_RM_ in a microglial nodule. Many CD3^+^ T cells are positive for CD103 (green arrow). Some CD103^+^CD3^+^ T cells also express CD69 (yellow arrow). CD49a, however is mostly expressed on endothelial cells of capillaries (magenta arrows), and only a minority of CD3^+^ T cells were positive for CD103, CD69, and CD49a. (d) Multiplex staining for CD3, CD8, CD103, CD69, and CD49a showing a blood vessel and surrounding parenchyma of a stage 2 lesion. Here, almost no CD103, CD69, or CD49a positive cells are present in the perivascular space (PS) of the blood vessel. CD69 expression on T cells in the parenchyma shows a similar distribution pattern to CD103^+^CD3^+^ T cells (T_RM_), although CD69 is generally weaker**. Yellow and green arrowheads depict two different CD103^+^CD69^+^CD3^+^ T_RM_, while the white arrowhead marks a CD103^+^CD49a^+^CD3^+^ T_RM_. The red insets show an enlargement of the CD103 (green arrowhead), CD69 (yellow arrowhead), or CD49a (white arrowhead) positive cells. Note that CD49a on this T_RM_ is very weak, whereas CD49a on capillaries (indicated with C) is much stronger. Data is indicated as median with interquartile range; PS= perivascular space, (**a-b**): Kruskal-Wallis test was performed, asterisk represents the result of post-hoc multiple comparison (Dunn’s test) *p ≤0.05

****Supplementary Figure 3: Expression of LAG-3 and PD-1****

(**a**) Density of PD-1^+^CD3^+^ T cells per mm^2^ across different stages. The numbers significantly increase with progression from stage 1 to stage 2 and stage 3-4. (**b**) The density of LAG-3^+^CD3^+^ T cells per mm^2^ also shows a significant increase from stage 1 to stage 2 and stage 3-4. (**c**) The proportion of CD8^+^CD3^+^ T cells expressing LAG-3 (as a percentage of all CD8^+^CD3^+^ T cells) across different stages. Here no significant stage-specific change was found. (**d**) The proportion of LAG-3⁺PD-1⁺CD3^+^ T cells as a percentage of all CD3^+^ T cells across disease stages. A significant increase from stage 1 to stage 3-4 was observed. (**e**) Venn diagram illustrating the proportion of CD3^+^ T cells expressing LAG-3, PD-1, or both markers. The data indicate that only 1.96% of all CD3⁺ T cells co-express LAG-3 and PD-1, while 14.69% express only PD-1, and 11.34% express only LAG-3 (median values are shown). (**f**) Percentage of PD-1^+^CD3^+^(yellow) and LAG-3^+^CD3^+^ T cells (red) among all CD3^+^ T cells during disease duration. No significant increase or decrease was detected (Spearman correlation p = 0.8584 and p = 0.5061, respectively). (**g**) Similarly, no correlation between the percentage of PD-1^+^CD8^+^CD3^+^(yellow) and LAG-3^+^CD8^+^CD3^+^ T cells (red) of all CD8^+^CD3^+^ T cells and disease duration was seen (Spearman correlation p = 0.6067 and p = 0.5169, respectively). (**a-d**) Kruskal-Wallis test was performed, asterisks represent the result of post-hoc multiple comparison (Dunn’s test) ***p ≤0.001, **p ≤0.01, *p ≤0.05; (**f**-**g**) Spearman’s rank correlation was performed.

Supplementary Figure 4: γδ^+^CD103^+^ T cells proliferate and show signs of exhaustion

(**a**) The density of γδ^+^CD3^+^ T cells in the parenchyma across different disease stages. More γδ^+^CD3^+^ T cells infiltrate the parenchyma in stage 2 and stage 3-4 in comparison to stage 1. (**b**) The proportion of CD103-expressing γδ^+^CD3^+^ T cells across disease stages. No significant differences were observed between stages 1, 2, and 3-4. (**c**) Comparison of the proportion of CD103-expressing γδ^+^CD3^+^ T cells in the parenchyma (P) versus perivascular space (PS) showed no significant differences. (**d)** Representative multiplex immunofluorescence images showing γδ^+^CD3^+^ T cells (red arrow) as well as CD8^+^CD3^+^ T cells (green arrow) expressing proliferation marker Ki67 (cyan) in a stage 2 lesion. (**e**) Here, instead of Ki67, we used the proliferation marker PCNA (yellow), indicating active proliferation of the CD8^+^ or γδ^+^ T cells. (**f**) The proportion of γδ^+^CD3^+^ T cells expressing PD-1 significantly increases in stage 3-4 compared to stage 1. (**g**) LAG-3 expression in γδ^+^CD3^+^ T cells remains consistent across stages. (**h-i**) Representative immunofluorescence images of γδ^+^CD3^+^ T cells expressing (**h**) PD-1 (yellow arrows) and (**i**) LAG-3 (cyan arrows) in a stage 2 lesion. (**a**-**c**, **f**-**g**) Kruskal-Wallis test was performed, asterisks represent the result of post hoc multiple comparison (Dunn’s test) ****p < 0.01**.

Supplementary Figure 5: Additional analysis of T_RM_ excluding the γδ^+^ T cell population

The analyses in Figures 2b-d were repeated with γδ⁺ T cells excluded. The overall trends and statistical significance remained unchanged, indicating that the results are not driven by the γδ T cell population: (**a**) The quantitative density of CD103^+^γδ^-^ CD3^+^ T cells (γδ^-^ T_RM_) across disease stages shows a significant increase of γδ^-^ T_RM_ from stage 1 to stage 2 and stage 3-4. (**b**) The proportion of γδ^-^ T_RM_ significantly increases from stage 1 to stage 3-4. (**c**) The percentage of γδ^-^CD3^+^ T cells expressing CD103 positively correlates with the amount of infiltrating γδ^-^CD3^+^ T cells (p = 0.0066). (**d**) No correlation between the percentage of γδ^-^ T_RM_ among all γδ^-^CD3^+^ T cells and disease duration is seen (p = 0.6031). (**a-b**) Kruskal-Wallis test was performed, asterisks represent the result of post-hoc multiple comparison (Dunn’s test) ***p ≤0.001, **p ≤0.01; (**c**-**d**) Spearman’s rank correlation was performed.
