# Supplementary figures and images for: The dynamics of brain T cell populations during the course of rasmussen encephalitis: from expansion to exhaustion
Source: J Neuroinflammation. 2025 Jun 12;22:155. doi: 10.1186/s12974-025-03477-5 (PMC12164096; doi:10.1186/s12974-025-03477-5)

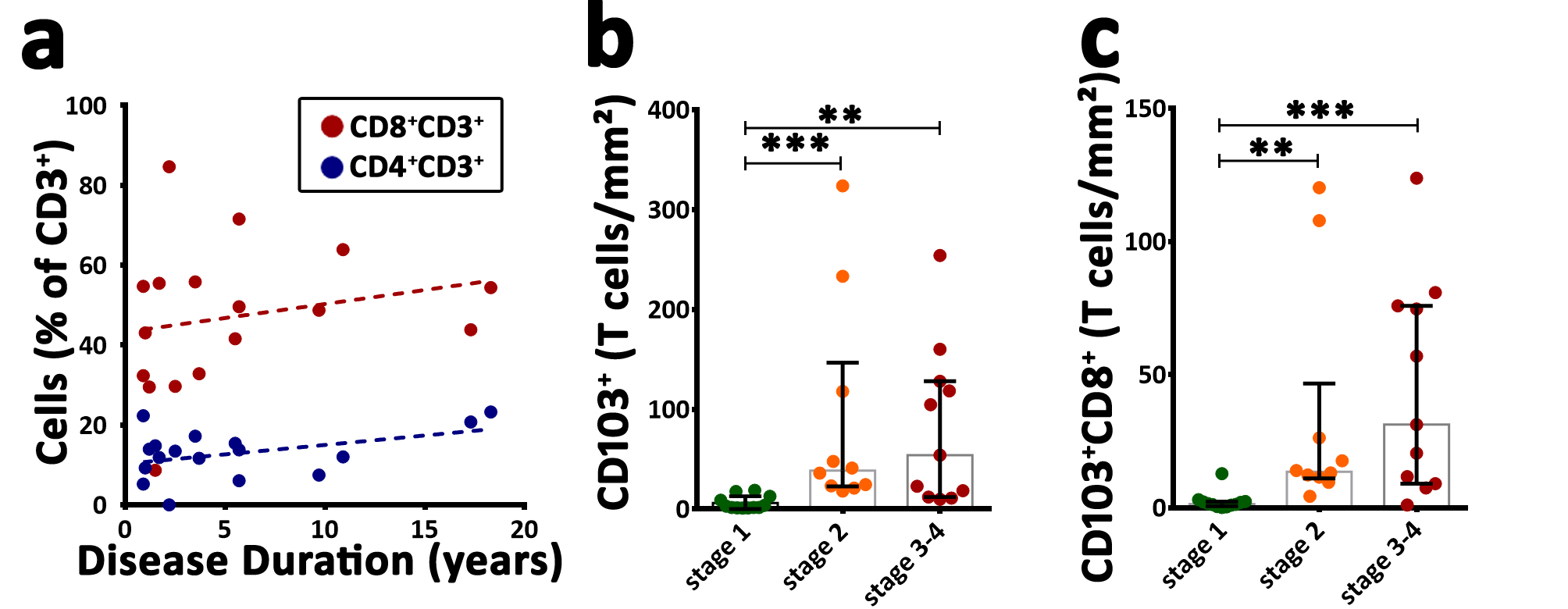

Supplement: Supplementary file 2 — Supplementary Material 2 [file 12974_2025_3477_MOESM2_ESM.jpg]

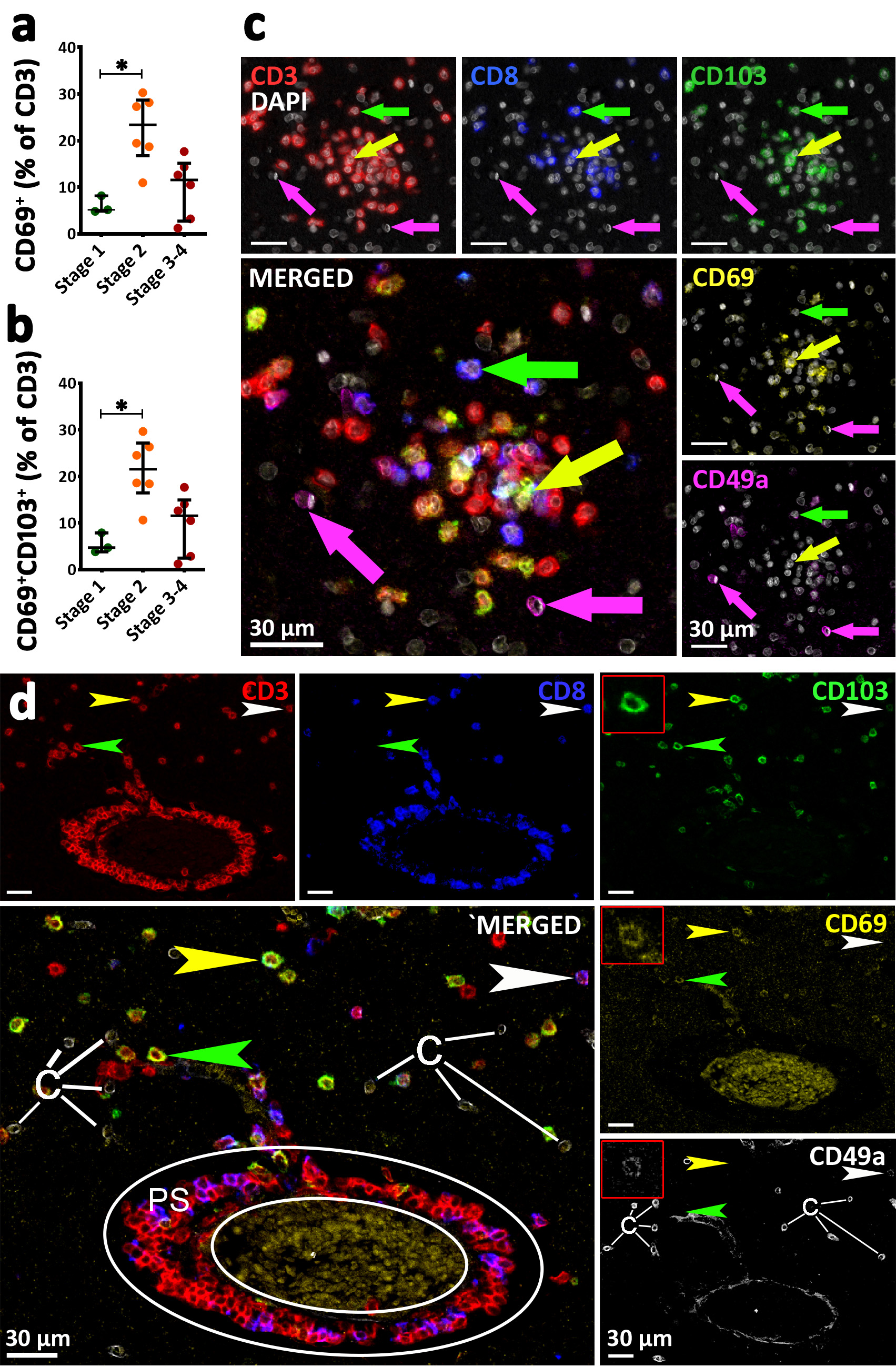

Supplement: Supplementary file 3 — Supplementary Material 3 [file 12974_2025_3477_MOESM3_ESM.jpg]

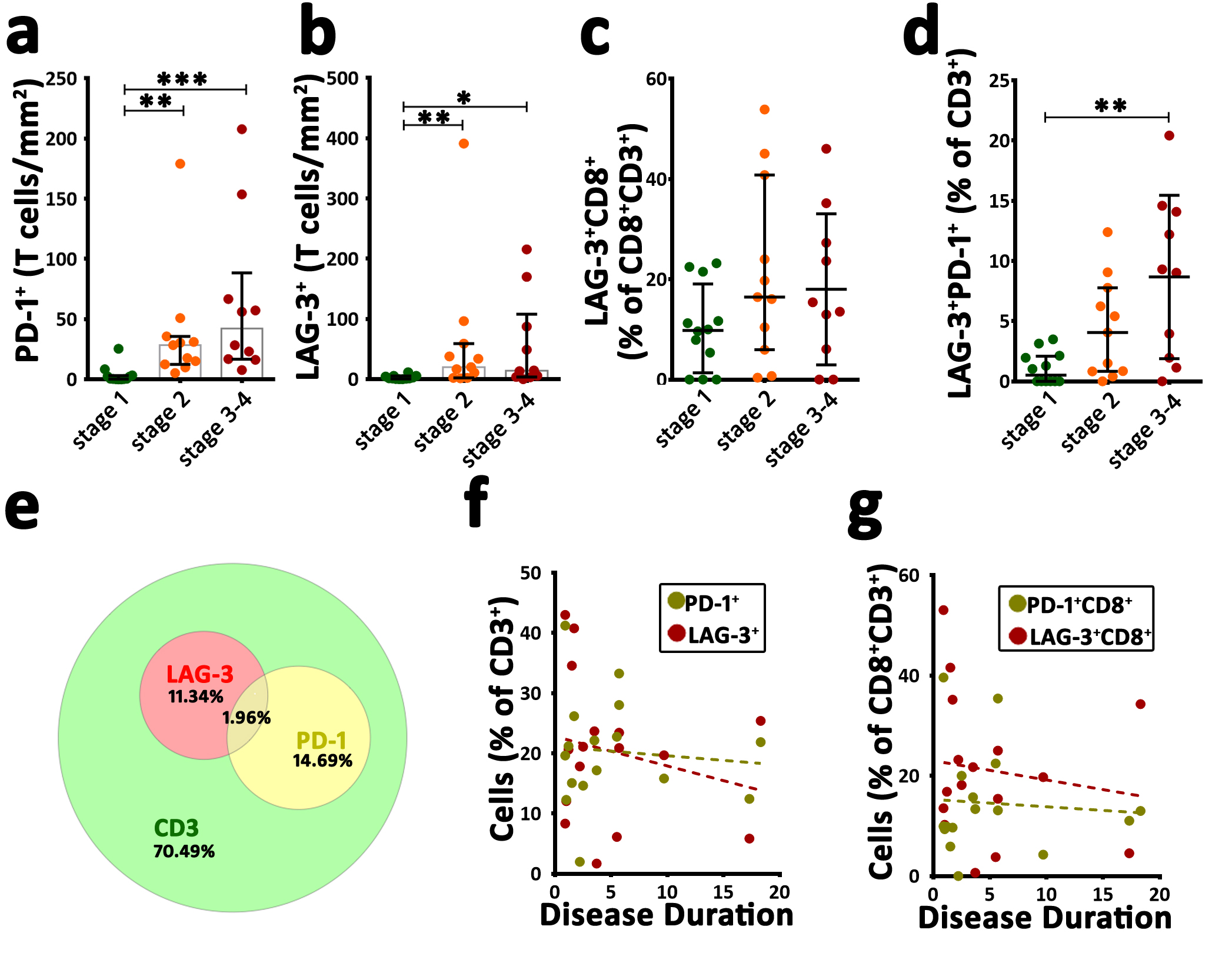

Supplement: Supplementary file 4 — Supplementary Material 4 [file 12974_2025_3477_MOESM4_ESM.jpg]

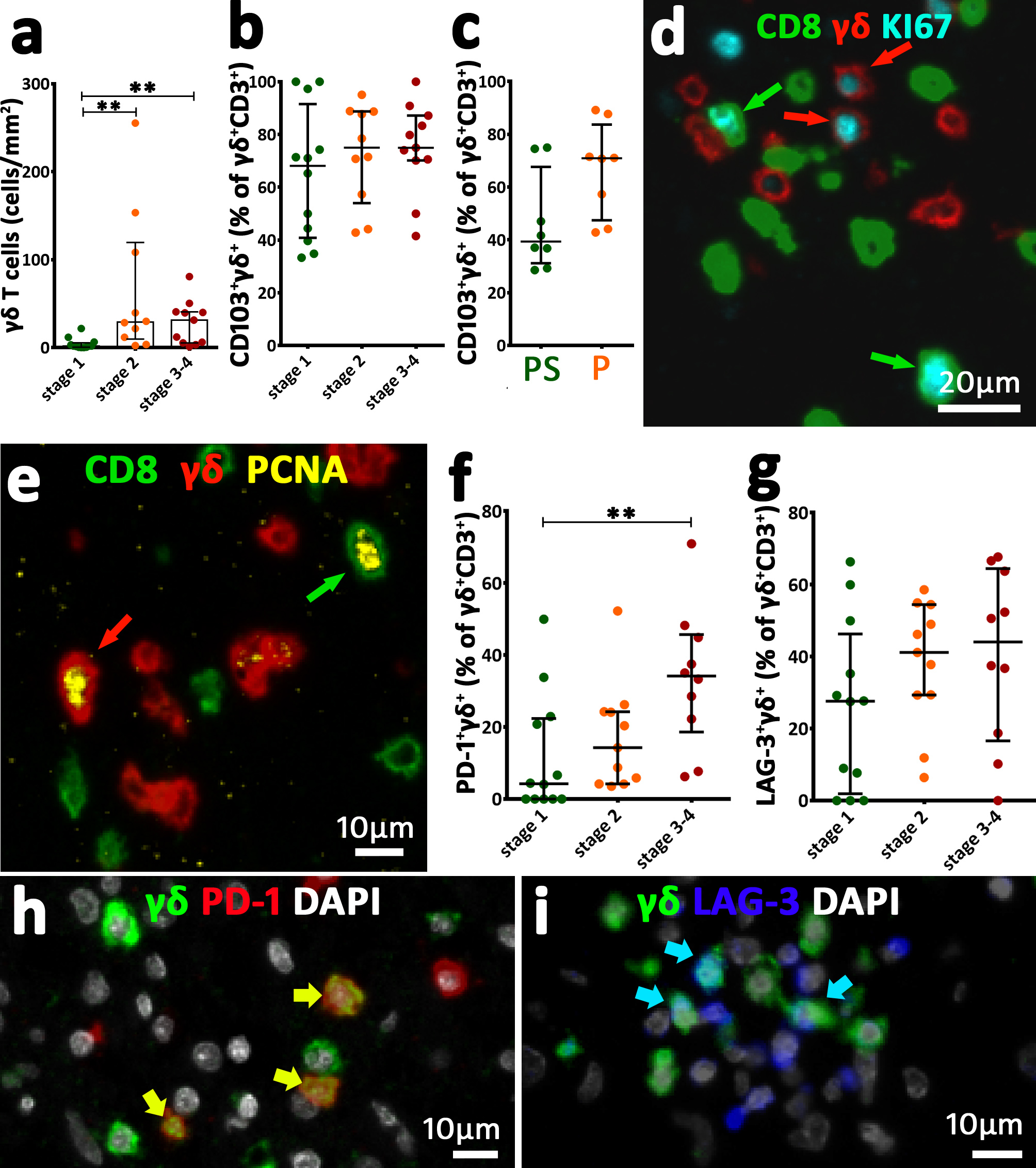

Supplement: Supplementary file 5 — Supplementary Material 5 [file 12974_2025_3477_MOESM5_ESM.jpg]

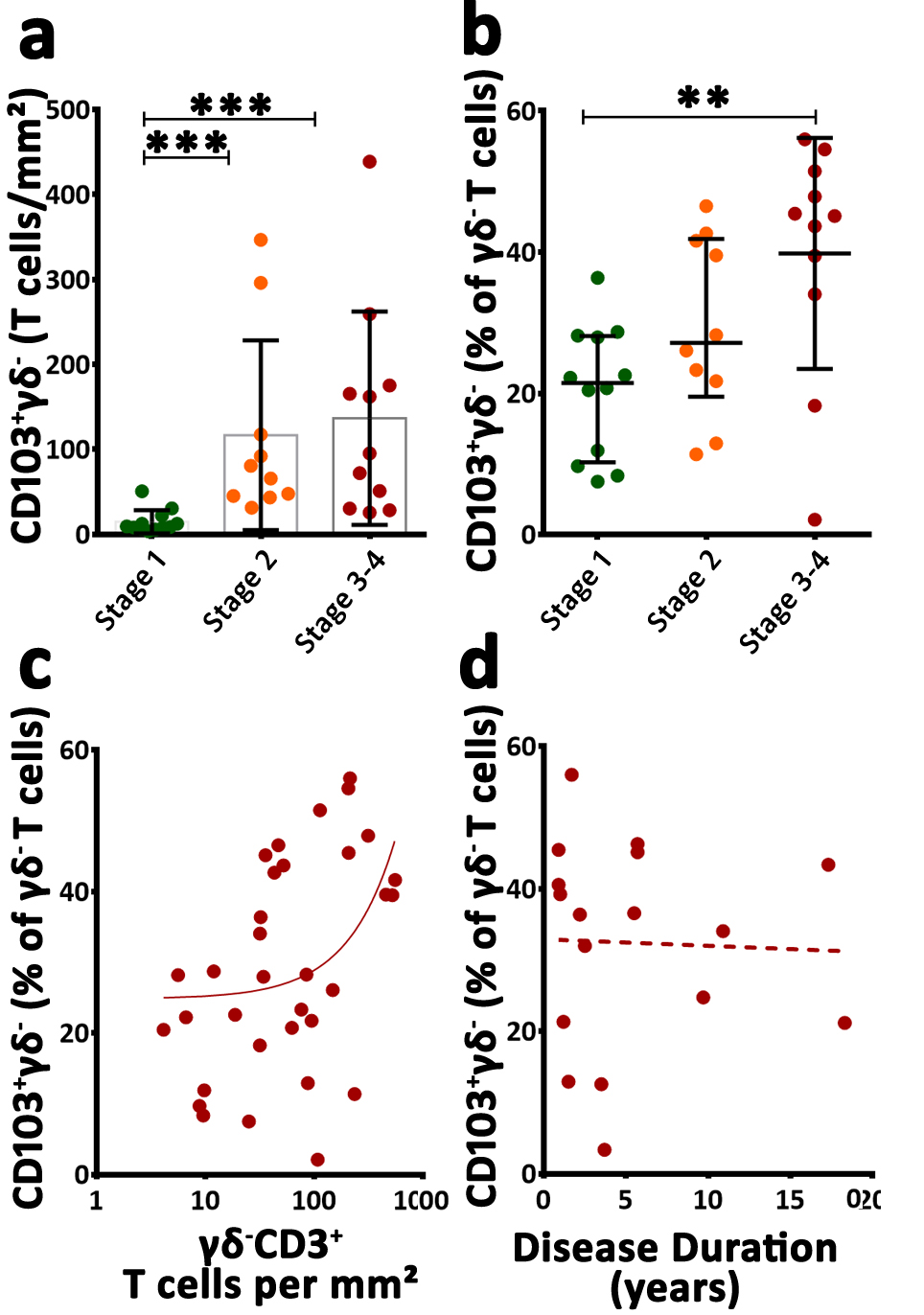

Supplement: Supplementary file 6 — Supplementary Material 6 [file 12974_2025_3477_MOESM6_ESM.jpg]
