# Supplementary material for: The dynamics of brain T cell populations during the course of rasmussen encephalitis: from expansion to exhaustion
Source: J Neuroinflammation. 2025 Jun 12;22:155. doi: 10.1186/s12974-025-03477-5 (PMC12164096; doi:10.1186/s12974-025-03477-5)
Supplement: Supplementary file 7 — Supplementary Material 7 [file 12974_2025_3477_MOESM7_ESM.docx]

| **Supplementary Table 1: Antibodies used for IHC and multiplex fluorescence** | | | | | | |
| --- | --- | --- | --- | --- | --- | --- |
| Antibody | Species | Target | Concentration | Secondary AB/Fluorophore | Pretreatment | Company |
| CD3 | Rabbit | epsilon chain of human CD3 | 1:500 +CSA enhancement 1.500 (Opal) | bi-α-rabbit^1^ pox-α-rabbit^2^ | EDTA pH9 AR9 | Neomarkers #RM9107-S |
| Iba1 | Rabbit | Microglia and Macrophage | 1:10000 | AP-α-rabbit^3^ | AR9 | Wako #019-19741 |
| HLA-DR | Mouse | HLA class II histocompatibility antigen DP, DQ, DRB1 (Human) | 1:100 | bi-α-mouse^4^ | EDTA pH9 | Dako  #M0775 |
| NeuN | Mouse | Vertebrate neuron-specific nuclear protein | 1:2500 +CSA enhancement 1:1000 | bi-α-mouse^4^ pox-α-mouse^5^ | Citrate | Chemicon #MAB377 |
| GFAP | Mouse | Glial Fibrillary Acidic Protein | 1:400 | AP-α-mouse^6^ | Citrate | Thermo Scient.  #MS-1376 |
| CD8 | Mouse | cytoplasmic domain of human CD8aα | 1:500 +CSA enhancement 1.500 (Opal) | bi-α-mouse^4^ pox-α-mouse^5^  Cy5-α-mouse ^4^ | EDTA pH9 AR9 | Dako #M7103 |
| CD4 | Mouse | endogenous levels of CD4 protein | 1:150 (Opal) | pox-α-mouse | AR9 | Cell Signaling  #48274 |
| CD103 | Rabbit | Human integrin α E | 1:5000 | pox-α-rabbit^2^ | AR9 | Abcam #ab129202 |
| CD69 | Rabbit | Human CD69 | 1:750 | pox-α-rabbit^2^ | AR9 | Invitrogen  #PA5-84010 |
| CD49a | Sheep | Human integrin α-1 | 1:74 | Cy2 Streptavidin^7^ | AR6 | Thermo Scientific  #PA5-47763 |
| CTLA-4 | Goat | *S. frugiperda* insect ovarian cell line Sf 21-derived recombinant human CTLA-4 | - | pox-α-goat | AR9 | R&D Systems  #AF-386-PB |
| LAG3 | Rabbit | Endogenous levels of total LAG3 protein | 1:300 | pox-α-rabbit^2^ | AR9 | Cell Signaling  #15372 |
| PD1 | Rabbit | human PD1 | 1:150 | pox-α-rabbit^2^ | AR9 | Abcam #137132 |
| TCR δ | Mouse | Human TCRδ protein | 1:10000 | pox-α-mouse^5^ | AR9 | SantaCru #sc-100289 |
| GrB | Mouse | Recombinant protein encoding the n-terminus of the mature granzyme B | 1:1000 +CSA enhancement 1:50 1:100 for Opal | bi-α-mouse^4^ pox-α-mouse^5^ | EDTA pH9 AR9 | Neomarkers #MS-1157-S1 |
| Ki-67 | Mouse | human recombinant peptide corresponding to a 1002 bp Ki-67 cDNA Fragment | 1:4000 | pox-α-mouse^5^ | AR9 | Dako #M7240 |
| PCNA | Mouse | Rat PCNA protein A fusion protein obtained from vector PC2T | 1:100000 | pox-α-mouse^5^ | AR9 | Dako  #M0879 |
| ^1^ 1:2000 Jackson immunoResearch #711-165-152  ^2^ 1:200 Jackson immunoResearch #711-035-152 ^3^ 1:100 Jackson immunoResearch #711-055-152 ^4^ 1:1000 Jackson immunoResearch #705-065-150 ^5^ 1:200 Jackson immunoResearch #715-035-151 ^6^: 1:100 Jackson immunoResearch#715-055-151 ^7^ 1:100 Jackson immunoResearch #016-220-084 | | | | | | |
