# Supplementary material for: The dynamics of brain T cell populations during the course of rasmussen encephalitis: from expansion to exhaustion
Source: J Neuroinflammation. 2025 Jun 12;22:155. doi: 10.1186/s12974-025-03477-5 (PMC12164096; doi:10.1186/s12974-025-03477-5)
Supplement: Supplementary file 8 — Supplementary Material 8 [file 12974_2025_3477_MOESM8_ESM.docx]

| **Supplementary Table 2: Multiplex Combinations** | | | | | |
| --- | --- | --- | --- | --- | --- |
| **Antibody** | **Opal dye/ fluorophore** | | | **Pretreatment** | |
| **T_RM_** staining | | | | | |
| α-CD103 | Opal 620 | | | AR9 1h | |
| α-CD69 | Opal 570 | | | AR6 30´ | |
| α-CD3 | Opal 690 | | | AR6 30´ | |
| α-CD8 | Opal 780 | | | AR6 30´ | |
| α-CD49a | Cy2 | | | AR6 30´ | |
| **γδ** staining | | | | | |
| α-TCR δ | Opal 570 | | | AR9 1h | |
| α-CD103 | Opal 620 | | | AR6 30´ | |
| α-CD8 | Opal 690 | | | AR6 30´ | |
| α-CD3 | Opal520 | | | AR6 30´ | |
| α-CD4 | Opal 780 | | | AR6 30´ | |
| **T_EX_** staining | | | | | |
| α-PD-1 | Opal 570 | | | AR9 1h | |
| α-LAG-3 | Opal 690 | | | AR6 30´ | |
| α-TCR δ | Opal 620 | | | AR6 30´ | |
| α-CD3 | Opal520 | | | AR6 30´ | |
| α-CD8 | Opal 780 | | | AR6 30´ | |
| **GrB/NeuN** staining | | | | | |
| α-NeuN | Opal 690 | | | Citrate 1h | |
| α-TCR δ | Opal 570 | | | AR9 45´ | |
| α-GrB | Opal 480 | | | AR6 30´ | |
| α-CD103 | Opal 620 | | | AR6 30´ | |
| **PCNA** staining | | | | | |
| α-PCNA | Opal 570 | | | - | |
| α-TCR δ | Opal 690 | | | AR9 1h | |
| α-CD8 | Opal 520 | | | AR6 30´ | |
| **KI69** staining | | | | | |
| Ki67 | Opal 690 | | | Citrate 1h | |
| α-TCR δ | Opal 520 | | | AR9 1h | |
| α-CD8 | Opal 620 | | | AR6 30´ | |
| **GrB** staining | | | | | |
| α-GrB | | Opal 520 | AR9 1h | |  |
| α-TCR δ | | Opal 570 | AR6 30´ | |  |
| α-CD103 | | Opal 690 | AR6 30´ | |  |
| α-CD3 | | Opal 780 | AR6 30´ | |  |
